# Benthic community succession on artificial and natural coral reefs in the northern Gulf of Aqaba, Red Sea

**DOI:** 10.1371/journal.pone.0212842

**Published:** 2019-02-27

**Authors:** Emily Higgins, Robert E. Scheibling, Kelsey M. Desilets, Anna Metaxas

**Affiliations:** 1 Department of Biology, Dalhousie University, Halifax, Nova Scotia, Canada; 2 Department of Oceanography, Dalhousie University, Halifax, Nova Scotia, Canada; University of Bremen, GERMANY

## Abstract

Evaluating the efficacy of artificial structures in enhancing or sustaining biodiversity on tropical coral reefs is key to assessing their role in reef conservation or management. Here, we compare spatial and temporal patterns of colonization and succession of the benthic assemblage on settlement collectors (ceramic tiles) in a 13-mo mensurative experiment on a suspended artificial reef, a seafloor artificial reef, and two nearby natural reefs at Eilat, Gulf of Aqaba. We also conducted a concurrent 7-mo manipulative experiment on the suspended reef and one of the natural reefs, and monitored fish feeding behaviour on experimental collectors, to examine effects of large mobile consumers on these patterns. In both experiments, taxonomic composition as percent planar cover for the whole community or biomass for the invertebrate component differed between collector topsides, dominated by a filamentous algal matrix, and shaded undersides with a profuse assemblage of suspension- or filter-feeding invertebrates. In the mensurative experiment, we found differences in final community and invertebrate composition between sites, which clustered according to reef type (artificial vs. natural) for collector undersides. Invertebrate biomass was greater at both artificial reefs than at one (undersides) or both (topsides) natural reefs. In the manipulative experiment, we found similar differences in composition between sites/reef types as well as between treatments (exclusion vs. control), and the invertebrate biomass was greater on the artificial reef. Invertebrate biomass was greater in the exclusion treatment than the control on collector undersides, suggesting mobile consumers can affect community composition and abundance. Predominant fish species observed interacting with collectors differed between artificial and natural reefs, likely contributing to differences in patterns of colonization and succession between sites and reef types. Our findings suggest artificial reefs have the potential to enhance cover and biomass of certain reef-associated assemblages, particularly those occupying sheltered microhabitats.

## Introduction

Coral reefs are deteriorating due to multiple natural and anthropogenic stressors [1–3]. Increasing ocean temperature and acidification, coupled with localized pollution, eutrophication, and harmful fishing practices, have accelerated the loss of structural and biological complexity on reefs worldwide [4–6]. Artificial reefs are being used increasingly to mitigate impacts on coral ecosystems. These reefs are human-made structures, sometimes with established coral colonies or fragments attached, intended to mimic natural reefs and enhance habitat availability for corals and reef-associated invertebrates and fish [7–9]. Specific conservation goals of artificial coral reefs include: restoration of 3-dimensional structure on degraded reefs [10]; enhancement of local biodiversity and survival [11–13]; accumulation of commercially important fish and invertebrates [12,14]; and provision of nursery sites for coral transplantation [15,16].

Algae and invertebrates typically colonize artificial reefs within 2–4 weeks of deployment [17–19]. Composition and abundance of the colonizing assemblage depend on reef size [18], proximity of source populations [20], local hydrodynamics [21], and composition [22,23] and orientation of the settlement surface [24–26]. The developing assemblage on artificial substrates can affect other reef-associated fauna, including fish and marine reptiles, although it may not resemble assemblages on adjacent natural reefs [12,27,28]. To assess the efficacy of artificial reefs as conservation tools, it is important to compare patterns and mechanisms of succession between artificial reefs and adjacent natural reefs.

On natural coral reefs, filamentous algae are early macroscopic colonizers in light-exposed areas, followed by crustose coralline algae, and fleshy and foliose brown algae [29–31]. Sessile suspension-feeding invertebrates typically recruit in shaded and sheltered microhabitats [19,32,33]. Reef-associated fishes and large mobile invertebrates, such as sea urchins and gastropods, can directly (as predators) or indirectly (as grazers) regulate recruitment and abundance of algae and sessile invertebrates [34,35]. Herbivorous fish play a key role in limiting algal biomass on coral reefs [27,36]: when abundant, they can denude the substratum of most algae [37]; when absent or at low abundance, filamentous turf-algae persists [17,38] and larger macroalgal forms proliferate [39,40].

Coral reefs in the northern Gulf of Aqaba, Red Sea, have long been exposed to natural and anthropogenic perturbations, including extreme low tide events, oil pollution, eutrophication, and diving tourism [41,42]. Conservation efforts in the region have included protection through marine reserves, and restoration using artificial reefs and coral transplantation [15,43]. These artificial reefs, which include oil platforms, shipwrecks, stone structures, and designed frameworks, can support vibrant and diverse fish and invertebrate communities [44] that in some cases surpass those on adjacent natural reefs in abundance [45]. Artificial reefs suspended above the seafloor have been deployed in the last two decades in the northern Gulf of Aqaba [46–48].

Suspended structures are a novel conservation tool in coral ecosystems in that they can be spatially isolated from natural reefs and localized stressors [49]. Benthic assemblages on suspended structures (e.g. pontoons) can differ from those on fixed structures (e.g. pilings, concrete blocks) or adjacent natural reefs [50–52], according to differences in environmental conditions related to height in the water column or isolation from the benthos [52,53]. Suspended artificial structures also may attract different fish species than natural reefs or structures on the seafloor [9,54], depending on their spatial orientation and height above bottom [55,56], habitat rugosity [57,58], and degree of fouling [59]. Suspended structures tend to attract more transient pelagic fish and fewer demersal fish than reefs on the seafloor [59].

Our study has two primary objectives. The first is to measure the pattern and rate of development of the benthic assemblage, on the exposed upper surface of settlement collectors (ceramic tiles) and the shaded/sheltered underside, in a mensurative experiment at four sites in the Gulf of Aqaba: a suspended artificial reef in open water, a seafloor artificial reef, and two natural reefs (one contiguous with the seafloor artificial reef). The second objective is to examine potential effects of grazing and predation by large mobile consumers on successional patterns and community development on the same type of collector in an exclusion (caging) experiment on the suspended artificial reef and one of the natural reefs. For the mensurative experiment, we predicted that the composition or abundance of colonizing invertebrates would not differ between reef sites, either on topsides or undersides of collectors, because the sites were in close proximity (< 7 km), collectors were deployed at a similar depth, and artificial and natural reefs harbored similar coral and invertebrate species. For the manipulative experiment, we predicted that abundance of algae and invertebrates would be greater in the exclusion treatment than the control at both sites, and that the accumulated biomass at the suspended reef (with fewer demersal herbivores) would exceed that at the natural reef. For both objectives, we predicted that different assemblages would develop between light-exposed and shaded microhabitats provided by our collectors, and that undersides would support a greater abundance of sessile invertebrates. Our overarching goal is to compare patterns and processes in the development of benthic assemblages on artificial and natural reefs to inform the use of artificial substrates as a potential mitigation tool for reef recovery through enhanced recruitment of corals and other invertebrates.

## Materials and methods

### Study sites and surveys of background community

**Fig 1:**
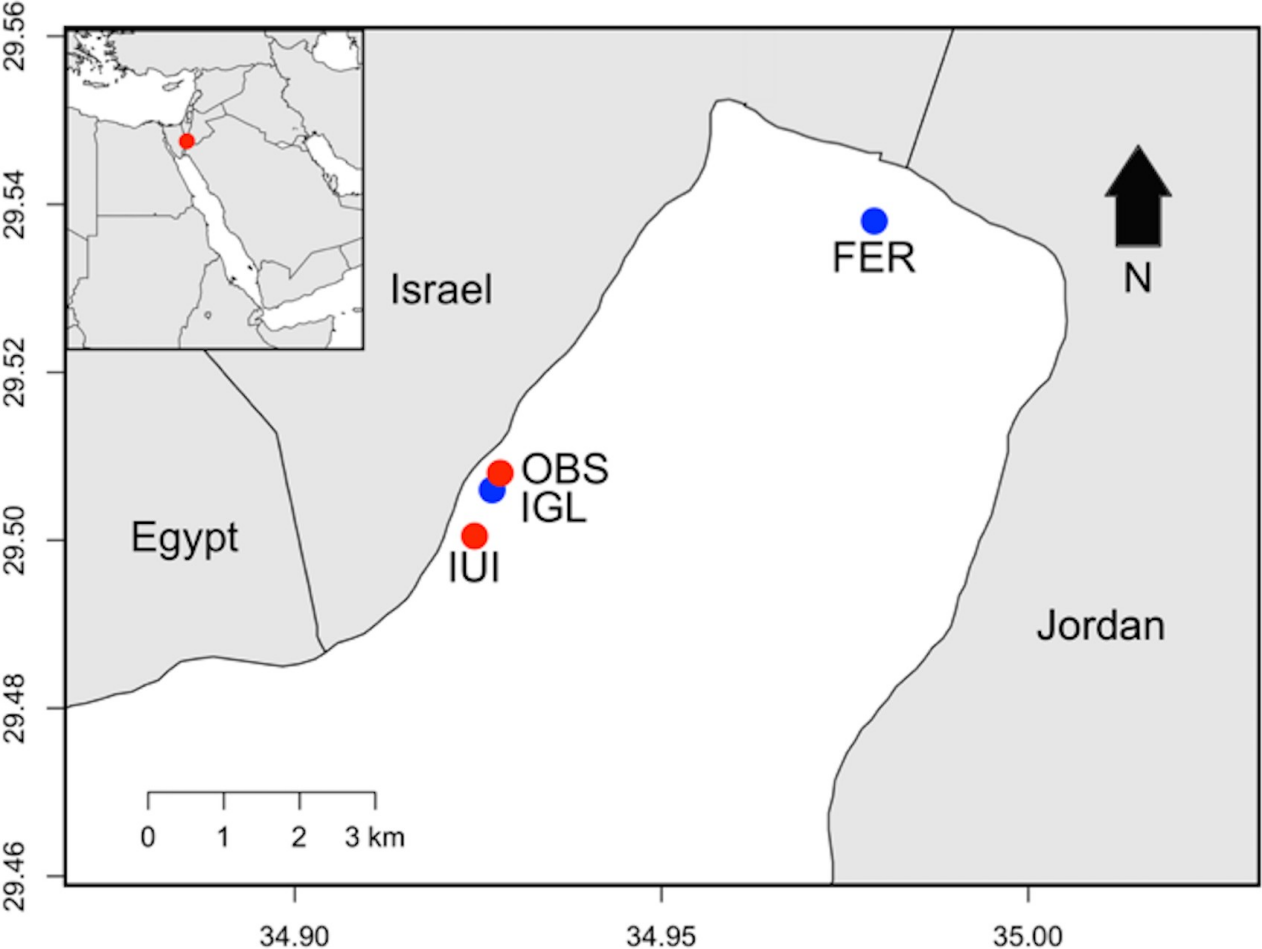
Map of Northern Gulf of Aqaba showing study sites. Suspended artificial reef (FER) and seafloor artificial reef (IGL) in blue, and two natural reef sites in red (IUI, OBS). Inset shows location of the Gulf in the Red Sea.

For the mensurative experiment, we measured colonization and succession of algae and invertebrates for 13 mo (October 2015–November 2016) on artificial collectors on a suspended artificial reef (Floating Experimental Reef, FER), a seafloor artificial reef (Igloo, IGL), and two natural reef sites (Interuniversity Institute Reef, IUI; Observatory Reef, OBS) in the northern Gulf of Aqaba. The manipulative experiment and concurrent fish observations were conducted over 7 mo (March 2016–November 2016) at FER and IUI. The structure at FER was deployed ~350 m off the northern shore of the Gulf (29°32’28.56”N, 34°58’25.38”E) in 2010 (Fig 1). It is suspended at 11-m depth on a framework (8 x 8 m) of large, air-filled polyethylene tubes. Plastic trays or mesh panels are attached to the upper surface of the suspended reef and contain transplanted and naturally recruited colonies of coral and other benthic invertebrates. The Igloo was deployed in 2001 within 20 m of OBS reef. It is a domed stainless-steel structure, 10 m in diameter and 3 m in height, at 10–13 m depth. The framework of the Igloo also is covered with transplanted corals and naturally recruited corals and other invertebrates. Study areas on natural reefs at IUI (29°30’03.45”N, 34°55’01.62”E) and OBS (29°30’12.50”N, 34°55’08.42”E) were constrained to the 10–13 m depth contour, comparable to the depth range of the two artificial reefs (Fig 1). The natural reefs are part of a fringing reef system in the Coral Beach Nature Reserve established in 1967.

To quantify the biotic assemblage on artificial and natural reefs at our study sites and provide context for our experiments, we photographically surveyed these areas using diver-operated cameras (Canon S100, Canon S110) in June 2015. The entire upper surface of FER was sampled with still photographs taken by divers swimming ~ 1 m above the reef platform. The shaded underside of the framework and cryptic microhabitats beneath it (undersides of plastic trays or the tubes that suspended the reef) were sampled similarly, using flash photography as necessary. At IGL, the entire outer surface was surveyed in a series of video transects conducted by divers swimming parallel to and at a constant distance from the evenly-spaced circular metal bands that supported the structure, from bottom to top. The underside of the IGL also was sampled systematically along these bands using flash photography. Structural elements on the artificial reefs provided scale for the photographs. At both IUI and OBS, a 100 x 0.8 m video belt-transect was conducted by divers swimming along the 10–13 m depth contour. A metal washer (4.5 cm in diameter) attached to a plumb line was used to maintain the camera at a fixed distance (2 m) above the seafloor and provide scale.

Videos were imported into GoPro Studio where contiguous, non-overlapping frames were extracted for each transect, and individual frame captures randomly selected to quantify percent cover of algae, stony corals, soft corals, and other sessile benthic invertebrates. Uncolonized frame area consisted of “bare” (without visually detectable fauna or flora) hard substratum (e.g. artificial structures, cobble, coral rubble and standing skeletons), sand (at natural reefs), and gaps (open water between artificial or biogenic structures at the artificial reefs). Frame captures on natural reefs (IUI: n = 12; OBS: n = 12) encompassed 28.8 m^2^ of the seafloor at each site. Still images of the upper platform (n = 30) and shaded underside (n = 30) at FER and frame captures of the outer surface (n = 30) and underside (n = 30) at IGL encompassed a total of 14.4 m^2^ and 4.8 m^2^ in reef surface area, for each side of each artificial reef, respectively. All images were analyzed using ImageJ64 (1.47v). Percent cover of taxonomic groups (see *Sampling and analysis of Experimental Collectors*) was calculated by overlaying 100 uniformly spaced points across the image. Points were disqualified if the substratum was obscured by a motile invertebrate.

Reef fish were opportunistically monitored by videography between 29 March and 19 April 2016 at FER, IGL and IUI. At each site, fixed cameras (GoPro Hero4, Canon S100, Canon S110, Canon G7X) on continuous video mode were trained (side view) for 20–40 min on collectors from the mensurative experiment (S1 Table). Video records were analyzed to identify fish species (presence/absence), record behaviour (grazing, foraging, egg aeration, territorial defense, sheltering), and measure feeding frequency (bite rates per video record, all species combined). The first and last minute of each video were removed as a buffer for potential effects of diver presence (divers moved out of sight of collectors during video sampling). Fish generally were observed to resume normal foraging behaviour around collectors within 1 min of a diver’s departure from an array once recording had begun, particularly those herbivorous fish with a greater potential for disturbance (e.g. parrotfish, surgeonfish). Preliminary analysis showed no significant difference in feeding frequency between video records with a 5-min vs. 1-min starting buffer, for each site (1-tailed, paired samples t-test: P > 0.43).

### Mensurative experiment

**Fig 2:**
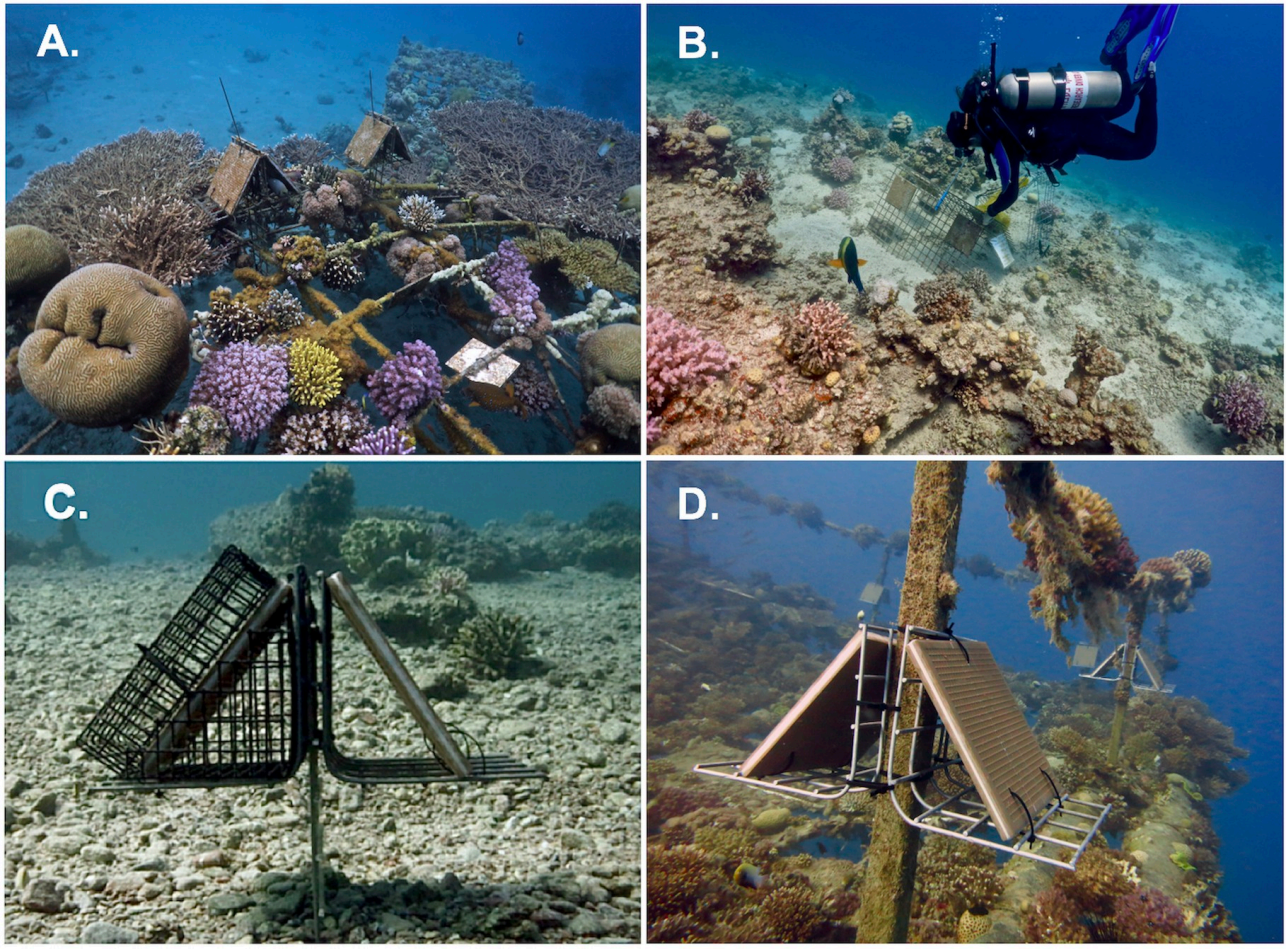
Experimental units and arrays at artificial and natural reefs. (A) 2-collector arrays at an artificial reef (IGL) and (B) a 4-collector array at a natural reef (IUI) in the mensurative experiment; (C) 2-collector arrays at a natural reef (IUI) in the manipulative experiment and (D) at an artificial reef (FER) in the mensurative experiment. Each collector (ceramic tile) in an array had 2 settlement surfaces (topside, underside). For the manipulative experiment (C), each array had 1 collector enclosed in a cage to exclude large consumers, the other collector served as an uncaged control.

For the mensurative experiment, settlement collectors were deployed at FER, IGL, IUI and OBS on 11–14 October 2015 (Permit no: 2015/41064). For each collector, 2 ceramic tiles (20 x 20 cm) were glued together with the unglazed sides exposed for settlement. This created 2 identical surfaces on each collector: a topside directly exposed to light and accessible to all consumers, and a sheltered and shaded underside. At the artificial reefs (FER and IGL), we attached paired collectors mounted at an angle of 45° (to limit sediment accumulation) on L-shaped frames of galvanized steel mesh and attached with plastic cable ties to form an array (Fig 2A). At each artificial reef, 10 arrays were dispersed around the upper surface of the structure at ~ 10 m depth and attached with cable ties to galvanized mesh (IGL) or metal posts (FER) to raise them 10–30 cm into the water column (Fig 2A). Due to permitting restrictions at IUI and OBS, single arrays included 4 collectors attached to large triangular frames of galvanized mesh (Fig 2B). Holes were cut out of the mesh to accommodate each collector. Five of these arrays were deployed at each site with 2 collectors spaced 35 cm apart and affixed to each side at a 45° angle. The apex of each array was 50 cm above the seafloor and the arrays were anchored on sandy bottom with buried concrete blocks. At each natural reef site, the arrays were spaced at 15–20 m-intervals along a 10–13 m-depth contour.

### Manipulative experiment

For the manipulative experiment, 8 replicate 2-collector arrays, similar to those used on artificial reefs in the mensurative experiment, were deployed at FER and IUI on 28 and 30 March 2016, respectively (Permit no: 2016/41336). For each array, 1 collector was enclosed in a cage to exclude large mobile consumers, while the other served as an uncaged control (Fig 2C). Black 16-gauge rigid wire mesh (1 x 2 cm aperture) was used to construct 4-cm high exclusion cages that enclosed the collector topside. Prism-shaped cages (triangular sides: base, 20 cm; height, 10 cm) were constructed to enclose the collector underside within the L-shaped mesh frame (Fig 2C and 2D). Due to spatial limitations on FER and permitting restrictions to the number of arrays at IUI (8), we elected to maximize replication of caged and control treatments by not including a procedural control (partial cage). To limit potential artefacts associated with biofouling, cages were replaced with cleaned ones at each sampling interval (monthly). On each side of the frame at FER, 2 units spaced 2–3 m apart were suspended from horizontal railings ~ 30 cm above the frame (Fig 2D). On each side, 1 array was randomly selected to orient the caged collector towards the center of the artificial reef and the control collector to face open water. At IUI, 8 arrays were secured 15–20 cm above the sand bottom on a vertical metal frame anchored with concrete bricks embedded in the sand (Fig 2C). The units were haphazardly positioned next to coral knolls at 10–13 m depth and spaced 2–4 m apart within a 90-m^2^ area, to provide a spatial layout comparable to that at FER.

### Sampling and processing of experimental collectors

For both experiments, topsides and undersides of collectors were photographically sampled at monthly intervals, using a metal framer fitted to the camera housing (Canon S100 with Fish Eye housing) to ensure the lens was centered and perpendicular to the collector surface at a fixed distance. To photograph the underside, divers cut a cable tie securing the collector to the top of the frame, allowing it to flip downwards. For caged collectors in the manipulative experiment, the upper cage was removed first to photograph the collector topside; the collector was then flipped to remove the lower cage and photograph the collector underside (Fig 2C and 2D). Cages were removed just prior to and replaced immediately after photographing the collector.

At the end of each experiment, all collectors were recovered to measure the composition and abundance of the attached invertebrate assemblage. Collectors from the mensurative experiment were recovered from FER, IGL, IUI, and OBS between 25 October and 15 November 2016. Collectors from the manipulative experiment were recovered from FER and IUI on 4 and 8 November 2016, respectively. Collectors from both experiments were photographed directly before collection (the final *in situ* photographic sample) and placed in pre-labelled Ziplock bags *in situ* for delivery to a research vessel where they were placed in large plastic bins, submerged in seawater and kept individually isolated and upright on an edge to prevent damage to assemblages on the surfaces. Within 20–40 min of recovery, collectors were transferred to flow-through seawater tables at the laboratory at the Interuniversity Institute, also isolated and upright, until processing.

Collectors were processed in random order within 5 d of recovery. Sessile macroinvertebrates (> 2 mm in maximum dimension) were manually removed by scraping with a scalpel under a dissecting microscope. Each individual was identified as a basal species/morphospecies or epibiont and photographed upon removal for future reference or identification. Algal films or turfs, encrusting sponges (≤ 3 mm thick), small oysters (< 5 mm), sessile polychaetes (< 1 cm) and coral recruits could not be effectively removed and their biomass was not recorded. In the mensurative experiment, cover of encrusting sponges was negligible or low on collector topsides at all reefs (< 1%) and undersides at natural reefs (< 4%), but formed a thin film on undersides at artificial reefs (17–23%; estimated biomass based on volume, 0.28–0.38 g). In the manipulative experiment, cover also was negligible or low on topsides and undersides in exclusion and control treatments at both the natural reef (< 4%) and artificial reef (3–8%). Therefore, exclusion of encrusting sponges did not appreciably underestimate our measures of sponge biomass. For the other taxa that could not be effectively removed from collectors, small individuals constituted ≤ 4% of oyster cover on artificial and natural reefs, and ≤ 3% and ≤ 1% of sessile polychaete cover on artificial and natural reefs, respectively. Coral recruits were measured (colony diameter, mm) and the number of polyps per recruit recorded.

Invertebrates were blotted on paper towel for ~ 5 min to remove excess water before biomass (wet weight, g 400 cm^-2^) was recorded on a top-loader electronic scale (precision, 0.01 g). Collectors were returned to flow-through seawater tables periodically during processing to prevent desiccation. Individuals were identified to the lowest taxonomic level by visual inspection in the laboratory during processing of collectors (S2 Table). A photographic catalogue was kept for all invertebrate taxa in both experiments. Samples of individuals that could not be identified to genus or species based on available taxonomic keys or local expertise were sent to experts for assistance.

For both experiments, photographs of individual collectors (image size: 4000 x 3000 pixels, 46 MB) from monthly samples were analyzed using ImageJ64 (1.47v) by overlaying 100 uniformly spaced points across the collector surface to measure percent planar cover of each taxonomic group of colonists. Points were excluded where the tile surface was obscured by a cable tie or motile invertebrate. Microbial or algal taxonomic groups included biofilms (bacteria, microalgae); algal matrix (conglomerate of filamentous turf-forming algae, sediment and detritus); macroalgae (non-coralline fleshy or foliose brown algae, e.g. *Lobophora* spp., *Padina* spp.); and encrusting coralline algae. Invertebrate groups included ascidians; bivalves; polychaetes; bryozoans; corals; and anemones. The only vertebrate group was damselfish eggs.

### Statistical analysis

Given the non-independence of exposed and cryptic surfaces on the same collectors, and expected large differences in assemblages between these surfaces, topsides and undersides of collectors were analyzed separately in both experiments. Given the difference in array structure between artificial and natural reefs in the mensurative experiment, we averaged the abundance (planar cover or biomass) of each taxonomic group across collectors (topside or underside) in each array (artificial reefs: n = 2 collectors; natural reefs: n = 4 collectors) for all analyses. We used PERMANOVA with Bray-Curtis similarity matrices to examine the effect of site (fixed factor, 4 levels: FER, IGL, OBS, IUI) in the mensurative experiment, and of site (fixed factor, 2 levels: FER, IUI) and treatment (fixed factor, 2 levels: exclusion, control) in the manipulative experiment, on the final composition of the entire assemblage (planar cover) and of invertebrate colonists (biomass). We used 1-way ANOVA to examine the effect of site or treatment on total invertebrate biomass or coral recruit density at the end of each experiment. We conducted post-hoc comparisons of sites (α = 0.05) using PERMANOVA pairwise t-tests (for the mensurative experiment) and Fisher's LSD test for ANOVA. We compared cover between adjacent exclusion and control treatments within experimental arrays using paired samples t-tests on selected sampling dates (when between-treatment differences in mean cover were greatest) for the manipulative experiment.

We examined temporal changes in composition of the assemblage (planar cover) during experiments, and differences in composition (planar cover, biomass) among sites or treatments at the end of experiments, using non-metric multidimensional scaling (nMDS) with Bray-Curtis similarity matrices. For the mensurative experiment, we used site means (average of array means within a site) to represent community composition on topsides or undersides at each time interval for each site for temporal analysis. For the manipulative experiment, we used treatment means (n = 8 collectors) to represent community composition on topsides or undersides at each time interval for each treatment at each site for temporal analysis. Analysis of similarity percentage (SIMPER) was used to identify taxonomic groups that contributed most to differences in composition between sites or treatments.

Planar cover of taxonomic groups was arcsine transformed and biomass of invertebrate groups was fourth-root transformed for PERMANOVA. PERMDISP test for PERMANOVA indicated that transformation succeeded in homogenizing variance (α = 0.05) for cover in both experiments and for biomass in the mensurative but not the manipulative experiment. Total invertebrate biomass and coral recruit density were log-transformed for ANOVA to satisfy Levene’s test for homogeneity of variance (α = 0.05). Minitab 18.1 statistical software was used for Levene’s test, ANOVA and post-hoc tests. PERMANOVA, PERMDISP, nMDS, and SIMPER analyses were computed in Primer v7.0 (Plymouth Routines in Multivariate Ecological Research) with PERMANOVA+ (PRIMER-E Ltd, Plymouth, UK).

## Results

### Community structure at artificial and natural reefs

At the start of our study in June 2015, average coral cover (stony and soft coral combined) on the substratum surface was 4- to 5-fold greater on artificial reefs (IGL, 43%; FER, 38%) than natural reefs (IUI and OBS, 9%) (S1 Fig). Stony corals dominated the cover at FER (32%), while soft corals dominated at IGL (24%). Stony corals also accounted for most of the coral cover at natural reefs (IUI, 8%; OBS, 6%). Cover of other sessile benthic invertebrates, including sponges, ascidians, bryozoans and bivalves, was low on the surface at artificial reefs (FER, 6%; IGL, 7%) and negligible at natural reefs. Macroalgae were observed only at FER (15%). Corals and other sessile invertebrates extensively covered the undersides of artificial reefs (FER, 84%; IGL, 59%) (S1 Fig). Coral cover (stony and soft coral combined) on undersides was 3-fold greater at IGL (44%) than FER (19%), with soft corals dominating at IGL (29%).

### Mensurative experiment

#### Cover of the colonizing community

**Fig 3:**
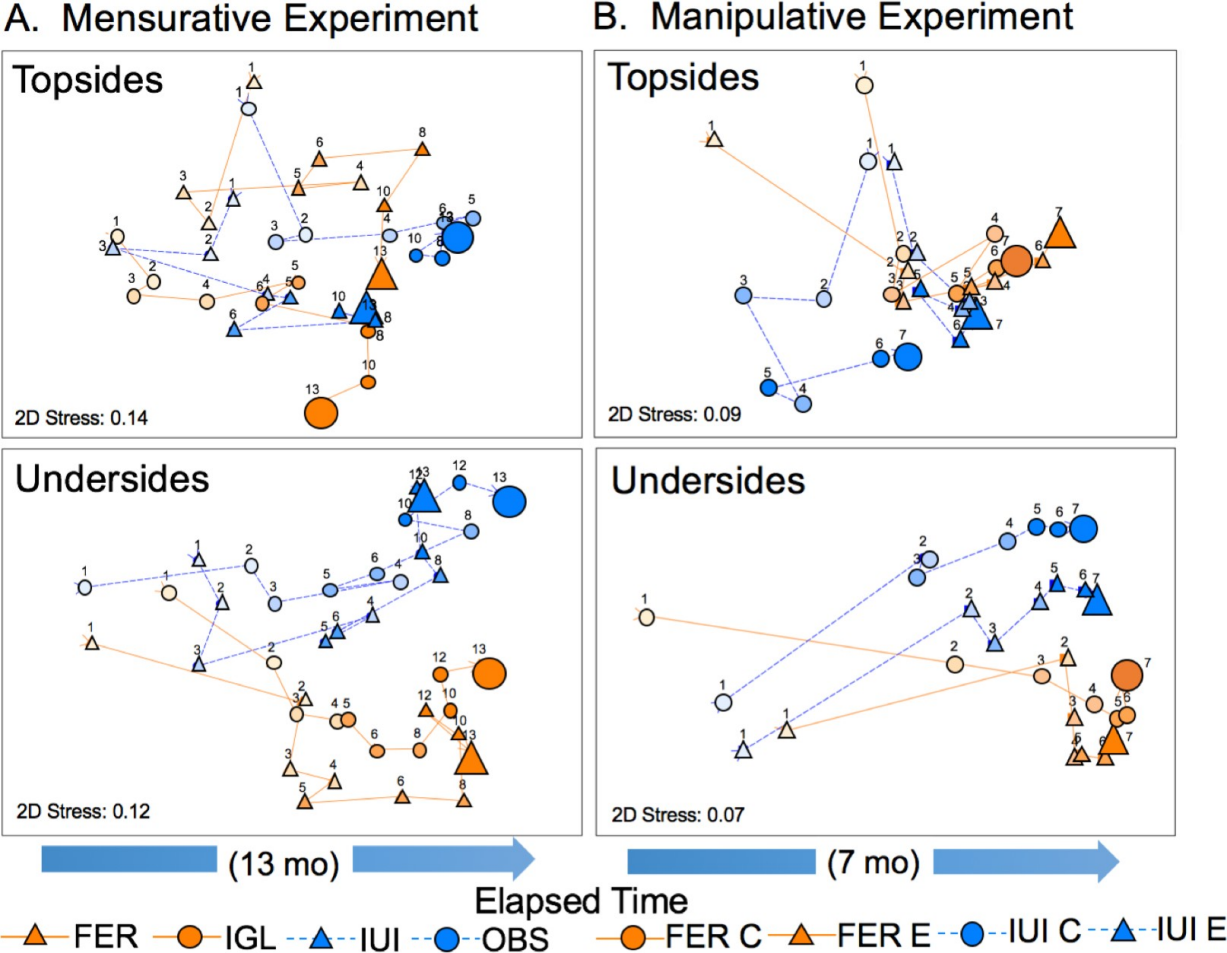
Trajectories of change in community composition among reefs in the mensurative and manipulative experiments. nMDS plot of community composition as planar cover (%) on collector topsides and undersides for (A) two artificial reefs (FER, IGL) and two natural reefs (IUI, OBS) over 13 mo in the mensurative experiment (Oct 2015–Nov 2016), and (B) for control (C) and exclusion (E) treatments at one of the artificial reefs (FER) and one of the natural reefs (IUI) over 7 mo in the manipulative experiment (Nov 2016). Each numbered symbol represents the average composition at that sampling interval (elapsed time, mo); symbol colour intensifies with time and symbols for final samples are magnified for clarity. Mensurative experiment: n = 10 arrays for artificial reefs, 5 arrays for natural reefs. Manipulative experiment; n = 8 collectors for each treatment x site combination.

On collector topsides, planar cover of the colonizing community on artificial and natural reefs was dominated by algal matrix and biofilm throughout the experiment (S2 Fig). Coralline algae increased in cover after 3 mo at IUI and IGL, respectively, but were rare at FER and OBS. Invertebrates colonized after 1 mo reaching maxima that were 1–2 orders of magnitude greater at artificial reefs than natural reefs. FER had the greatest cover of invertebrates, mainly polychaetes (from 2–6 mo), bryozoans, and bivalves (from 6–13 mo). Invertebrates were rare at IGL until 8 mo, when sponges and bivalves colonized. At natural reefs, invertebrates were rare throughout the experiment. nMDS showed that trajectories of change in community composition on topsides were most divergent at FER and OBS, while trajectories at IGL and IUI converged after 4 mo, when collectors were colonized by coralline algae (Fig 3A).

On collector undersides, invertebrates began colonizing earlier at artificial reefs (after 2–3 mo) than natural reefs (after 4–5 mo) and maintained a greater total cover at artificial reefs throughout the experiment (S2 Fig). Greatest planar cover was observed at artificial reefs by bBBBryozoans between 3 and 6 mo, and ascidians at 8 mo. In contrast, at natural reefs, a greater cover of biofilm and algal matrix remained throughout the experiment, peak cover of bryozoans was lower, and bivalves appeared earlier and reached greater peak cover (S2 Fig). nMDS showed that trajectories of change in community composition were similar between sites within reef types; reef types diverged after 2–3 mo (Fig 3A).

**Fig 4:**
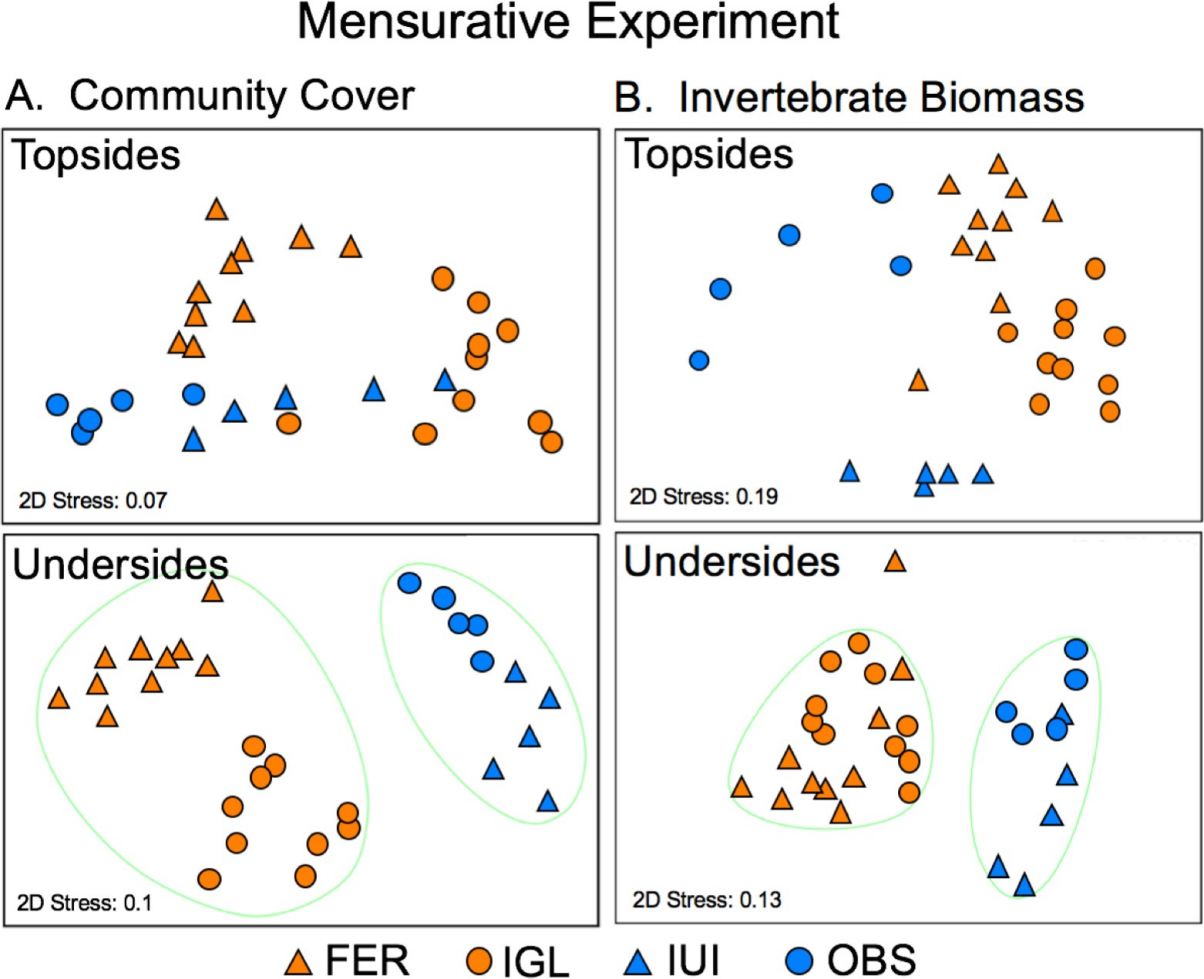
Final community composition on artificial and natural reefs in the mensurative experiment. Non-metric multidimensional scaling (nMDS) plot of (A) community composition as planar cover (%) and (B) invertebrate composition as biomass (g 400 cm^-2^) on collector topsides and undersides (averaged per array) at two artificial reefs (FER, IGL; orange symbols, n = 10 arrays) and two natural reefs (IUI, OBS; blue symbols, n = 5 arrays) at the end of the mensurative experiment (Nov 2016). Ellipses bound groups of arrays of 70% similarity.

PERMANOVA showed that community composition (arcsine-transformed data) differed significantly (P < 0.001) between sites on both topsides and undersides at the end of the 13-month experiment; pairwise comparisons indicate that all sites differed from one another (P < 0.01) in both cases (S3 Table). Coralline algae and algal matrix accounted for most of the dissimilarity in community composition on topsides between sites, while bivalves, bryozoans and sponges accounted for most of the dissimilarity on undersides (S4 Table). nMDS showed that arrays on artificial and natural reefs formed separate clusters at 70% similarity for undersides, but were more interspersed between reef types for topsides (Fig 4A).

#### Final biomass of invertebrate colonists

**Fig 5:**
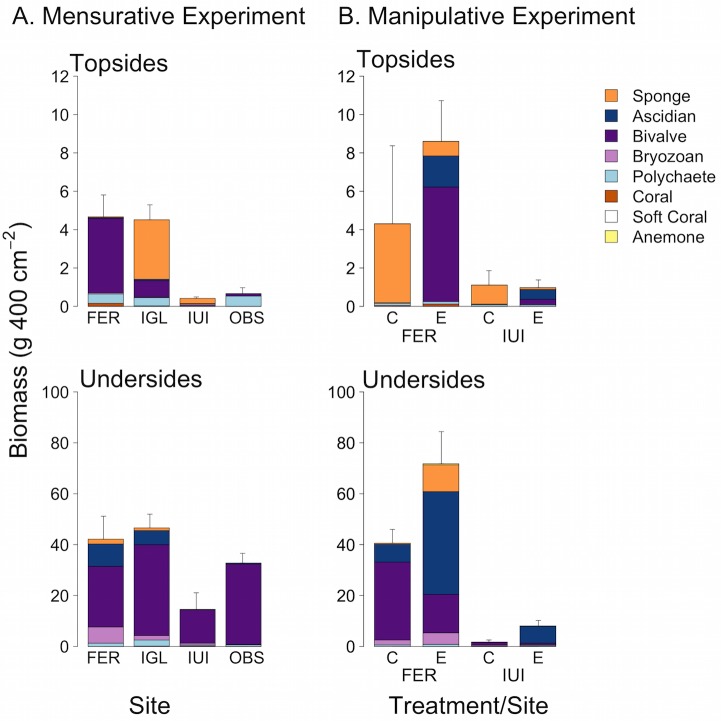
Final biomass of invertebrates on artificial and natural reefs in the mensurative and manipulative experiments. Biomass (g 400 cm^-2^) of invertebrate colonists on collector topsides and undersides for (A) two artificial reefs (FER, IGL; n = 10 arrays) and two natural reefs (IUI, OBS; n = 5 arrays) at the end of the mensurative experiment (Nov 2016), and for (B) control (C) and exclusion (E) treatments at one of the artificial reefs (FER) and one of the natural reefs (IUI) (n = 8 collectors for each site x treatment combination) at the end of the manipulative experiment (Nov 2016). Bar heights are mean (+SE) total biomass for arrays in the mensurative experiment and collectors in the manipulative experiment.

Total biomass was an order of magnitude greater on undersides than topsides across all sites at the end of the experiment (Fig 5A). ANOVA showed that biomass (log-transformed data) on topsides differed among sites (F_3,26_ = 12.9, P < 0.001) and was greater on artificial reefs than natural reefs, but sites within each reef type did not differ (Fisher’s LSD test, α = 0.05). For undersides, ANOVA yielded a marginally non-significant result for the effect of site (F_3,26_ = 2.96, P = 0.051), but pairwise comparisons showed that FER, IGL and OBS formed one homogeneous subset, and OBS and IUI another, indicating that biomass at IUI but not OBS was significantly lower than that at the two artificial reefs.

Sponges, bivalves, and ascidians accounted for most of the macroinvertebrate biomass on topsides and undersides of collectors at the end of the experiment (Fig 5A). PERMANOVA showed that the composition of invertebrates, as for the entire community, also differed between sites (P < 0.001) and in all pairwise combinations (P < 0.01) on both topsides and undersides of collectors by the end of the experiment (S5 Table). Sponges, bivalves and polychaetes accounted for most of the dissimilarity in composition on topsides between sites, while ascidians, bivalves and bryozoans accounted for most of the dissimilarity on undersides (S6 Table). nMDS showed that arrays on artificial and natural reefs also formed separate clusters for undersides, with all but 1 array (at FER) clustering at 80% similarity by reef type, but tended to cluster more by site for topsides, with the greatest dissimilarity among arrays at OBS (Fig 4B).

### Manipulative experiment

#### Cover of the colonizing community

On collector topsides, planar cover of the colonizing community was dominated by algal matrix and biofilm throughout the experiment in exclusion and control treatments at both sites (S3 Fig). Cover of invertebrates in both treatments was low and consisted mainly of sponges and ascidians. Fleshy macroalgae were absent on collector topsides and encrusting coralline algae occurred only at IUI. At FER, patches of damselfish (*Neopomacentrus miryae*, *Dascyllus trimaculatus*) eggs appeared on control topsides at 1 and 4 mo. At IUI, total cover on topsides was significantly greater in exclusion than control treatments between 3 and 5 mo (paired t-test, P < 0.01), whereas a wheret FER total cover was significantly greater in the control than exclusion treatment at 1 mo (P = 0.035) (S3 Fig). Total cover in the exclusion treatment at both sites generally exceeded 80% by 3 mo. nMDS showed that trajectories of change in community composition in control and exclusion treatments on topsides were more divergent at IUI than at FER (Fig 3B).

The community that developed on undersides of collectors differed from that on topsides: invertebrates (mainly ascidians, oysters, bryozoans, sponges and polychaetes) were much more abundant, particularly at FER (S3 Fig). At FER, ascidians reached maximum cover by 3 mo, but then declined as individuals senesced and the cover of oysters, bryozoans and sponges increased. At IUI, controls were largely covered by biofilm and algal matrix until 5 mo, and cover of invertebrates increased after 4 mo as cover of biofilm declined. Damselfish also laid eggs in control treatments at the start of the experiment at IUI (*Pomacentrus trichrourus*, 1–3 mo) and FER (*Neopomacentrus xanthurus*, *N*. *miryae* 1–2 mo). Total cover on undersides was significantly greater in the control than the exclusion treatment at 2 and 3 mo at IUI (paired t-test, P < 0.04) and at FER at 1 mo (P < 0.001), mainly due to damselfish eggs and biofilm. In contrast, total cover was significantly greater in the exclusion treatment at 2 and 3 mo at FER (P < 0.003), mainly due to ascidians. Total cover in both treatments exceeded 60 and 80% by 4 mo at IUI and FER, respectively. nMDS showed that trajectories of change in community composition in control and exclusion treatment diverged after 3 mo, reflecting a greater increase in abundance of invertebrates at FER (Fig 3B).

**Fig 6:**
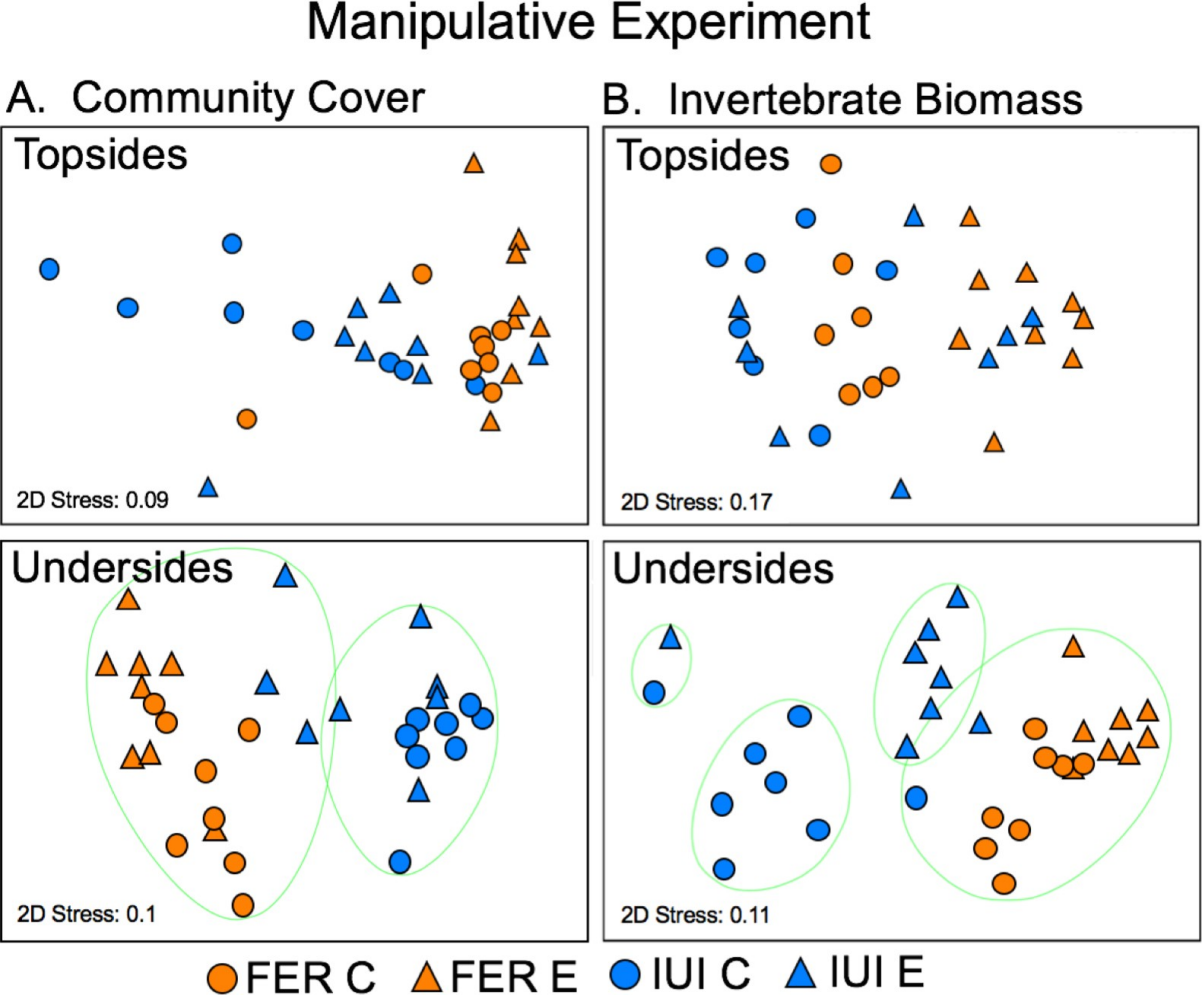
Final community composition in treatments at reefs in the manipulative experiment. Non-metric multidimensional scaling (nMDS) plot of (A) community composition as planar cover (%) and (B) invertebrate composition as biomass (g 400 cm^-2^) on collector topsides and undersides in control (C, circles) and exclusion (E, triangles) treatments (n = 8 collectors) at an artificial reef (FER, orange symbols) and a natural reef (IUI, blue symbols) at the end of the manipulative experiment (Nov 2016).

At the end of the manipulative experiment, community cover on collector topsides was dominated by algal matrix in both treatments at both sites, with a low abundance of invertebrates (mainly sponges) at FER (S3 Fig). In contrast, a diverse assemblage of invertebrates dominated the undersides of collectors, particularly at FER where they had displaced algal matrix and biofilm (S3 Fig). PERMANOVA showed that community composition differed significantly (P < 0.001) between sites on both topsides and undersides, and between treatments on undersides but not topsides (P = 0.076) (S7 Table). For topsides, algal matrix, biofilm, and sponges accounted for most of the dissimilarity between sites; for undersides For ost ocmpariascidians, biofilm, algal matrix, and bryozoans accounted for most of the dissimilarity between treatments within sites and between sites within treatments (S8 Table). nMDS showed that both control and exclusion treatments separated by site on undersides, but less so on topsides (Fig 6A).

#### Final biomass of invertebrate colonists

Mean total biomass was an order of magnitude greater on the FER than the IUI for both treatments on undersides and for the exclusion treatment on topsides, and 4-fold greater for the control on topsides but not statistically significant (t_14_ = 2.74, P = 0.395), at the end of the experiment (Fig 5B). Total biomass on topsides did not differ significantly (paired t-test) between exclusion and control treatments at either site (IUI: t_7_ = -0.162, P = 0.562; FER: t_7_ = 0.925, P = 0.193). In contrast, total biomass on undersides was significantly greater in the exclusion treatment than the control at both sites (IUI: t_7_ = 2.78, P = 0.014; FER: t_7_ = 2.35, p = 0.026).

As in the mensurative experiment, sponges, bivalves, and ascidians accounted for most of the macroinvertebrate biomass on topsides and undersides of collectors at the end of the manipulative experiment (Fig 5B). PERMANOVA showed that the composition of invertebrates differed significantly (P < 0.001) between sites and between treatments for both topsides and undersides, with a significant interaction between factors (P = 0.048) for undersides only (S9 Table). Ascidians and bivalves accounted for most of the dissimilarity in community composition between treatments and sites on topsides; ascidians, bivalves, and sponges accounted for most of the dissimilarity between treatments within sites and between sites within treatments on undersides (S10 Table). nMDS showed that both control and exclusion treatments generally separate by site and treatment on undersides, less so on topsides (Fig 6B).

### Recruitment of stony corals

Stony corals accounted for < 3.4% and < 1% of planar cover in the mensurative and manipulative experiments, respectively. Coral recruits at the end of each experiment (n = 220 and 108 respectively) were almost exclusively *Stylophora* spp. with one *Seriatopora* sp. recruit in each experiment and one *Leptastrea* sp. recruit in the mensurative experiment. Recruits ranged in size from 2.0 to 20.7 mm with 4 to 119 polyps. In the mensurative experiment, mean recruit density ranged from 0.4–2.2 recruits 400 cm^-2^ on topsides and varied significantly between sites (ANOVA, F_3,26_ = 3.35, P = 0.034); pairwise comparisons (Fisher’s LSD test, α = 0.05) showed that FER and IUI formed one homogeneous subset, and IUI, IGL and OBS another, indicating that recruit density was greatest at FER and lowest at IGL and OBS (S4A Fig). Mean recruit density was more variable on undersides (0.1–4.9 recruits 400 cm^-2^) and differed significantly between sites (ANOVA, log x+1-transformed data, F_3,26_ = 51.4, P < 0.001); pairwise comparisons (Fisher’s LSD test, α = 0.05) showed density at IUI > OBS > IGL = FER (S4A Fig). In the manipulative experiment, the range of mean recruit density on topsides and undersides was similar to those recorded in the mensurative experiment. Recruit density was significantly greater at FER than IUI on topsides (2-way ANOVA, log x+1 transformed data, F_1,28_ = 4.7, P = 0.038) but greater at IUI than FER on undersides (F_1,28_ = 31.7, P < 0.001) (S4B Fig). Recruit density was significantly greater in the control than exclusion treatment on undersides (F_1,28_ = 5.1, P = 0.032) but not topsides (F_1,28_ = 1.2, P = 0.280). The interaction of site and treatment, for both topsides or undersides, had no significant effect on coral recruit density (P > 0.8).

### Fish activity

Mean feeding frequency by fish on collector topsides at FER, IGL and IUI ranged from 0.2 to 0.8 bites min^-1^ (S11 Table) and did not differ between sites (ANOVA, F_2,38_ = 0.95, P = 0.396). Feeding on undersides was observed only at IUI (S11 Table) and did not differ in frequency from that on topsides at this site (paired t-test, t_16_ = -1.17, P = 0.261). Fish species observed biting included purple-brown parrotfish (*Scarus fuscopurpureus*), striated surgeonfish (*Ctenochaetus striatus*) and brown surgeonfish (*Acanthurus nigrofuscus*) at IUI, and striated surgeonfish, rusty parrotfish (*Scarus ferrugineus*) and yellowtail tang (*Zebrasoma xanthurum*) at IGL (S12 Table). Red Sea goatfish (*Parupeneus forsskali*) were foraging on the collectors with their barbels at IGL, but were not observed biting. Damselfish (*Neopomacentrus miryae*, *Dascyllus trimaculatus*) were observed biting on topsides at FER where they engaged in algal gardening as well as territorial defense and egg aeration. Damselfish (*N*. *miryae*, *N*. *xanthurus*, *D*. *trimaculatus*) and other small fish, such as sea goldies (*Pseudanthias squamipinnis*), blennies and wrasses were observed sheltering on collector undersides at FER and IGL (S12 Table).

## Discussion

### Benthic community development and accumulated biomass on artificial and natural reefs

The succession of algae and sessile invertebrates and accumulated cover and biomass after 13 mo differed between reef types (artificial, natural) depending on collector aspect (topside, underside). This is contrary to our prediction of a null effect, i.e. that communities would evolve similarly between reef types at the same depth and with similar source populations of algae and invertebrates in the surrounding area. On collector undersides, community composition according to cover began to diverge between artificial and natural reefs after 3 mo in both experiments. By the end of the experiments, sites segregated by reef type in terms of the composition of both cover and accumulated invertebrate biomass. Differences in composition between reef types were less clear on topsides, where there were significant differences among all sites. Our findings suggest that when free space is made available on heavily colonized artificial reefs, the emergent benthic assemblages can differ in composition and abundance from those on natural reefs. This pattern is particularly pronounced when artificial reefs are elevated (IGL) or suspended (FER) off bottom and have luxuriant communities of filter- and suspension-feeding invertebrates (e.g. ascidians, bivalves, bryozoans, and sponges) growing on their undersides, where increased flow and reduced sedimentation are conducive to growth and reproduction of these “fouling species” [21,60].

As expected, community composition and invertebrate biomass in both experiments differed greatly between collector topsides, dominated largely by a filamentous algal matrix, and shaded and sheltered undersides that accumulated a diversity of sessile benthic invertebrates. Similar differences in composition and abundance of colonists, depending on collector aspect, are well documented in previous studies on temperate [26,61] and tropical reefs [19,45,62]. Light exposure is the key proximate factor accounting for these differences [25,63,64]. Rapid growth of a dense algal matrix on collector topsides may have inhibited settlement of invertebrate larvae through preemption of space [65,66] or chemical deterrence [67,68]. An algal matrix also traps sediment that can smother recruits of filter- and suspension-feeding invertebrates [69], and erect macroalgae can overgrow recruits of corals [67,70] and ascidians [71]. Competitive interactions among fast-growing algal and invertebrate species result in high species turnover [19]. Our findings suggest that artificial reefs that are designed with expanses of shaded, cryptic microhabitats can foster growth of fouling species, such as sponges, bivalves, bryozoans, and ascidians.

Patterns of succession within reef types and collector aspects differed between sites despite their proximity (meters to 7 km) and similar depth (10–13 m). Hydrodynamic models of the Gulf of Aqaba describe a chain of gyres [72,73], with the northern section of the gulf dominated by a single gyre throughout the year [74]. This circulation pattern may result in high larval retention [75,76], likely from a single larval pool within our general study area, which would lead to similar rates of larval supply to all reefs. This process can explain the relatively high cover of bivalves across all our reef sites. However, recruitment rates of some fouling species, such as ascidians and sponges, can vary at small spatial scales because their larvae are short-lived (minutes to days) resulting in low dispersal potential (10s – 100s m) [77–80]. These taxa, together with bryozoans, constituted a significant component of the recruits on our collectors and of the fouling communities on the structural undersides of IGL and the FER. Our results suggest that fouling communities on artificial reefs could be maintained through self-recruitment to the reef.

### Exclusion of mobile consumers

As we predicted, cages that excluded large mobile consumers significantly altered community composition and abundance of sessile invertebrates on collectors in the manipulative experiment, particularly on collector undersides. At both FER and IUI, the accumulated biomass on undersides at the end of the experiment was significantly greater in the exclusion treatment than in the control, with ascidians and sponges accounting for most of the between treatment difference in the composition. Predation and grazing can be important causes of mortality of ascidian recruits [71,81] and chemically undefended sponges [82,83], and could account in large part for these differences between treatments. We are unable to unambiguously test for predation effects because potential artifacts of caging were not measured within our constrained experimental design. Common artifacts include reduction of light and water flow; both of these factors may enhance settlement of ascidian larvae [81]. However, given the large mesh size of our cages (1 x 2 cm aperture), possible artifactual effects were likely small compared to the consumptive effects of fish and sea urchins [84], particularly on sheltered undersides with relatively low levels of ambient light and flow.

Differences in fish assemblages and types of herbivores (scrapers, grazers, croppers) between the artificial and natural reefs in our study likely contributed to differences in patterns of succession and accumulated abundance among sites. Suspended artificial reefs limit access by demersal fish that frequent artificial reefs on the seafloor or natural reefs [53,59]. The FER harbours an abundant and diverse assemblage of planktivorous fish, but demersal herbivorous fish (croppers) rarely were observed. In contrast, the fish assemblage at IGL and the two natural reefs also includes piscivores and demersal carnivores and herbivores [85]. Territorial damselfish can be important herbivores on reefs where roving foragers are not present [86]. Observations of biting on collectors by scarids (mostly scrapers) and acanthurids (grazers) at IGL suggest that sessile invertebrate assemblages on seafloor artificial reefs may be exposed to similar grazing pressure as on natural reefs; we found that IGL and IUI followed similar trajectories of succession over time on exposed topsides of collectors in the mensurative experiment.

Differences in array design between artificial and natural reefs may have influenced the size of mobile consumers that could access collector undersides. The restricted space within the L-shaped frame of the 2-collector arrays, used at IGL and FER in the mensurative experiment (Fig 2A and 2D), and at FER and IUI (Fig 2C) in manipulative experiment, likely restricted foraging by large fish on collector undersides compared to the 4-collector arrays on larger, angled mesh stands at OBS and IUI (Fig 2B). This is consistent with the absence of feeding observations (biting) on collector undersides in video records of 2-collector arrays in the mensurative experiment at IGL and FER, although small fishes (e.g. blennies and damselfish) occasionally were observed swimming through or briefly sheltering beneath the collectors. The latter generally were planktivorous species compared to the larger grazers and scrapers (e.g. surgeonfish, parrotfish) that had access to collector topsides at all sites in both experiments and to undersides at IUI and OBS in the mensurative experiment. Fish feeding frequency did not differ between topsides and undersides on the 4-collector arrays at IUI.

Diadematid sea urchins are important grazers on coral reefs worldwide and their effects on turf algal abundance and coral recruitment have been documented in a previous caging experiment at IUI [84]. *Diadema setosum* settled and grew on the suspended tubular structure of the FER, but recruits were sparsely distributed. Individuals ranging from 1 to 6 cm diameter occasionally were recorded on collectors during the mensurative (8 cases) and manipulative (2 cases, control treatment) experiments at FER, but not at IGL or our natural reef sites, although they occurred in small aggregations in cryptic microhabitats on patch reefs in shallower areas.

### Recruitment of stony corals

Despite their abundance and diversity in the background communities on natural and artificial reefs, the cover of stony corals was low (< 4%) and almost exclusively *Stylophora* spp. in both our experiments. Recruit size (2–21 mm diameter) at the end of the experiments in November 2016 suggests these corals settled between April and August, based on growth rates in the Gulf of Aqaba [87]. Recruit density generally was ≤ 2 recruits 400 cm^-2^ across sites, collector aspects, and treatments, except on undersides at IUI where it reached 4–5 recruits 400 cm^-2^ in both experiments. This is comparable to recruit density previously measured on ceramic [87] and PVC [45] recruitment plates in the region. Abelson et al. [88] documented lower recruitment rates of stony corals (< 1 recruit 400 cm^-2^), which were attributed to low survival rates and high abundances of fouling species at experimental sites. We also observed the lowest levels of coral recruitment on undersides of artificial reefs, possibly due to the dense cover of fouling species (mainly ascidians and sponges) that preempted space or inhibited recruitment through allelochemical interactions [89–91]. In contrast, cover on the undersides at the IUI, where coral recruitment was greatest, was dominated by bivalves, algal matrix and biofilm. Recruit density was greater in the control than exclusion treatment on undersides in the manipulative experiment, possibly due to grazing by sea urchins [84]. Given the relatively slow growth of corals, multi-year studies are required to detect the establishment of adult coral colonies and their eventual dominance on experimental structures [92].

## Conclusions

Our experimental results underscore the potential of artificial reefs to enhance cover and biomass of reef-associated assemblages, particularly those occupying sheltered microhabitats. The assemblages of algae and invertebrate colonists that developed on standardized collectors on artificial reefs diverged after 4 months from those on natural reefs and accumulated more invertebrate taxa with greater total biomass. Throughout both experiments, light-exposed and shaded surfaces of collectors supported distinct benthic communities: algae dominated the cover on topsides while dense fouling communities developed on undersides. Differences between artificial reef sites in the mensurative experiment, including composition of the resident community, platform structure and elevation above bottom, and proximity to the natural reefs, likely contributed to variation in community composition and accumulated biomass between sites.

Both experiments were relatively short (7 and 13 months), making it difficult to discern differences due to seasonality versus length of deployment [93–95]. For example, seasonal variation in spawning and recruitment of invertebrate colonizers may have produced different results had the experiments been deployed at different times of year [88]. The short duration of the experiments also limits our ability to assess stony coral recruitment, as we only captured a single recruitment event. Although all sites have a common larval pool for invertebrates with long larval duration, observed differences in community composition of colonists between artificial and natural reef sites likely are due to variation in the local reef assemblage, particularly among sessile invertebrates with short larval duration or resident populations of fish and invertebrate predators that can limit recruitment.

Our findings highlight the importance of strategic design considerations in the deployment of artificial reefs to achieve conservation, management or economic objectives, including: 1) architecture of the physical platform and its elevation above bottom, 2) population of the structure with transplanted colonies of corals and other invertebrates, and 3) positioning of the artificial reef relative to neighboring natural reefs and propagule sources. An added advantage of suspended artificial reefs, anchored to the seabed, is that of vertical positioning in the water column to optimize the establishment and growth of targeted reef-associated species, and the potential to alter horizontal or vertical position to avoid detrimental changes in environmental conditions (e.g. warming, anoxia) or pulsed disturbances (e.g. pollution events, flash floods, strong storms). In the face of increasing pressures on coral reefs, artificial reefs are being proposed as one mechanism for remediation; however, the variation we observed in our study suggests that the desired conservation outcome needs to be well defined to ensure success of such mitigation efforts.

## Supporting information

S1 Fig Background community composition on artificial and natural reefs. Mean (+SD) planar cover (%) and composition of sessile benthic organisms on upper (top) surface (FER_T_, IGL_T_: n = 30 frames) and underside (FER_U_, IGL_U_: n = 30 frames) of the platform on artificial reefs, and on upper surface of natural reefs (IUI_T_, OBS_T_: n = 12 frames), in June 2015.
(TIF)

S2 Fig Spatial and temporal patterns in community composition on artificial and natural reefs in mensurative experiment. Change in planar cover (%) and composition of taxonomic groups on collector topsides and undersides at a suspended artificial reef (FER), a seafloor artificial reef (IGL), and 2 natural reefs (IUI, OBS) over 13 mo in the mensurative experiment (Oct 2015–Nov 2016). Bar heights are mean (+SE) of 10 arrays (2 collectors averaged per array) for artificial reefs and 5 arrays (4 collectors averaged per array) for natural reefs at each sampling interval.
(TIF)

S3 Fig Temporal patterns in community composition between treatments and reefs in the manipulative experiment. Change in planar cover (%) and composition of taxonomic groups on (A) collector topsides and (B) undersides for exclusion (E) and control (C) treatments at a suspended artificial reef (FER), and a natural reef (IUI) during the 7-mo manipulative experiment (April 2016–Nov 2016). Bar heights are mean (+SE) of 8 collectors for each treatment at each sampling interval. Asterisks indicate intervals when control and exclusion treatments are significantly different (α = 0.05).
(TIF)

S4 Fig Final coral density on artificial and natural reefs in the mensurative and manipulative experiments. Mean (+SE) density of stony coral recruits (individuals 400 cm^-2^) on collector topsides and undersides for (A) two artificial reefs (FER, IGL; n = 10 arrays) and two natural reefs (IUI, OBS; n = 5 arrays) at the end of the mensurative experiment (Nov 2016), and for (B) control (C) and exclusion (E) treatments at one of the artificial reefs (FER) and one of the natural reefs (IUI) (n = 8 collectors for each site x treatment combination) at the end of the manipulative experiment (Nov 2016).
(TIF)

S1 Table Video records to identify fish species and record behaviour. Metadata from dives between 27 Mar and 19 Apr 2016 at a suspended artificial reef (FER), a seafloor artificial reef (IGL), and a natural reef (IUI).
(DOCX)

S2 Table Taxonomic identification of colonizing species in mensurative and manipulative experiment. Data are the lowest taxonomic level (Cl, Class; Or, Order; Fa, Family) identified for invertebrates on collector topsides (T) and undersides (U) at the end (Nov 2016) of a 13-mo mensurative experiment at a suspended artificial reef (FER), a seafloor artificial reef (IGL), and two natural reefs (IUI, OBS), and a 7-mo manipulative experiment at two of these sites (FER, IUI) for exclusion (E) and control (C) treatments.
(DOCX)

S3 Table PERMANOVA of community composition in the mensurative experiment. Analysis examines the effect of site (fixed factor: FER, IGL, IUI, OBS) on the composition of planar cover (%) on topsides and undersides of collectors at the end of the 13-mo experiment. Also shown are pairwise comparisons using the PERMANOVA t-statistic. Tests are based on 999 permutations. Significant results in bold.
(DOCX)

S4 Table SIMPER analysis of community composition in the mensurative experiment. Analysis indicates the contribution of different taxonomic groups to dissimilarity in the composition of planar cover (%) between combinations of sites (FER, IGL, IUI, OBS), for topsides and undersides of collectors, at the end of the 13-mo experiment.
(DOCX)

S5 Table PERMANOVA of invertebrate composition in the mensurative experiment. Analysis examines the effect of site (fixed factor: FER, IGL, IUI, OBS) on the composition of invertebrate biomass (g 400 cm^-2^) on topsides and undersides of collectors at the end of the 13-mo experiment. Also shown are pairwise comparisons using the PERMANOVA t-statistic. Tests are based on 999 permutations. Significant results in bold.
(DOCX)

S6 Table SIMPER analysis of invertebrate composition in the mensurative experiment. Analysis indicates the contribution of different taxonomic groups to dissimilarity in the composition of invertebrate biomass (g 400 cm^-2^) between combinations of sites (FER, IGL, IUI, OBS), for topsides and undersides of collectors, at the end of the 13-mo experiment.
(DOCX)

S7 Table PERMANOVA of community composition in manipulative experiment. Analysis examines the effects of site (FER, IUI) and treatment (exclusion, control) on the composition of planar cover (%) on topsides and undersides of collectors at the end of the 7-mo experiment. Significant results in bold.
(DOCX)

S8 Table SIMPER analysis of community composition in the manipulative experiment. Analysis indicates the contribution of different taxonomic groups to dissimilarity of the composition of planar cover (%) between treatments (exclusion, E; control, C) at an artificial (FER) and natural (IUI) reef, and between reefs for each treatment, on topsides and undersides of collectors at the end of the 7-mo experiment.
(DOCX)

S9 Table PERMANOVA of invertebrate composition in the manipulative experiment. Analysis examines the effects of site (FER, IUI) and treatment (exclusion, control) on the composition of invertebrate biomass (g 400 cm-2) on topsides and undersides of collectors at the end of the 7-mo experiment. Significant results in bold.
(DOCX)

S10 Table SIMPER analysis of invertebrate composition in the manipulative experiment. Analysis indicates the contribution of different taxonomic groups to dissimilarity of the composition of invertebrate biomass (g 400 cm^-2^) between treatments (exclusion, E; control, C) at an artificial (FER) and natural (IUI) reef, and between reefs for each treatment, on topsides and undersides of collectors at the end of the 7-mo experiment.
(DOCX)

S11 Table Feeding frequency of fish on artificial and natural reefs. Data are feeding frequency (bites min^-1^) for all fish species on collector topsides and undersides at a suspended artificial reef (FER), a seafloor artificial reef (IGL), and at a natural reef (IUI) based on video records from 29 March and 19 April 2016 (S1 Table). Data are mean ± SE for video records (n) in which fish were observed biting the collectors.
(DOCX)

S12 Table Fish species and activities on artificial and natural reefs. Species identification and observed behaviour on collector topsides and undersides at 3 sites in the mensurative experiment (artificial reefs at FER and IGL, natural reef at IUI) from all video records. Species: *Scarus fuscopurpureus*, *Scarus ferrugineus*, *Ctenochaetus striatus*, *Acanthurus nigrofuscus*, *Zebrasoma xanthurum*, *Parupeneus forsskali*, *Heniochus diphreutes*, *Cyclichthys spilostylus*, *Ecsenius gravieri*, *Neopomacentrus miryae*, *Neopomacentrus xanthurus*, *Dascyllus trimaculatus*, *Pseudanthias squamipinnis*. Behaviour: biting (B), foraging (F), egg aeration (A), defending territory (D), sheltering (S).
(DOCX)
