# Supplementary figures and images for: Benthic community succession on artificial and natural coral reefs in the northern Gulf of Aqaba, Red Sea
Source: PLoS One. 2019 Feb 27;14(2):e0212842. doi: 10.1371/journal.pone.0212842 (PMC6392313; doi:10.1371/journal.pone.0212842)

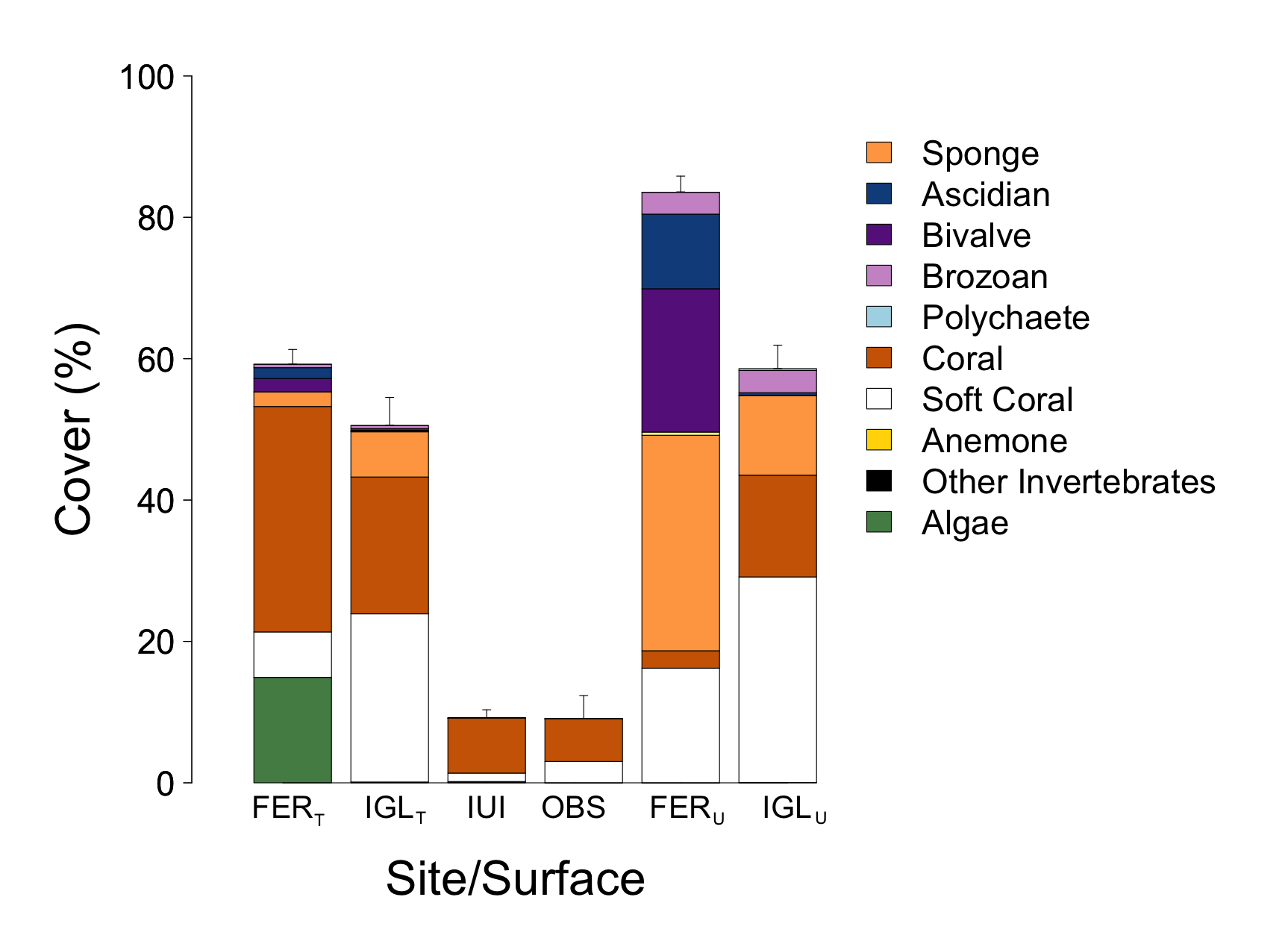

Supplement: S1 Fig — Mean (+SD) planar cover (%) and composition of sessile benthic organisms on upper (top) surface (FERT, IGLT: n = 30 frames) and underside (FERU, IGLU: n = 30 frames) of the platform on artificial reefs, and on upper surface of natural reefs (IUIT, OBST: n = 12 frames), in June 2015. (TIF) [file pone.0212842.s001.tif]

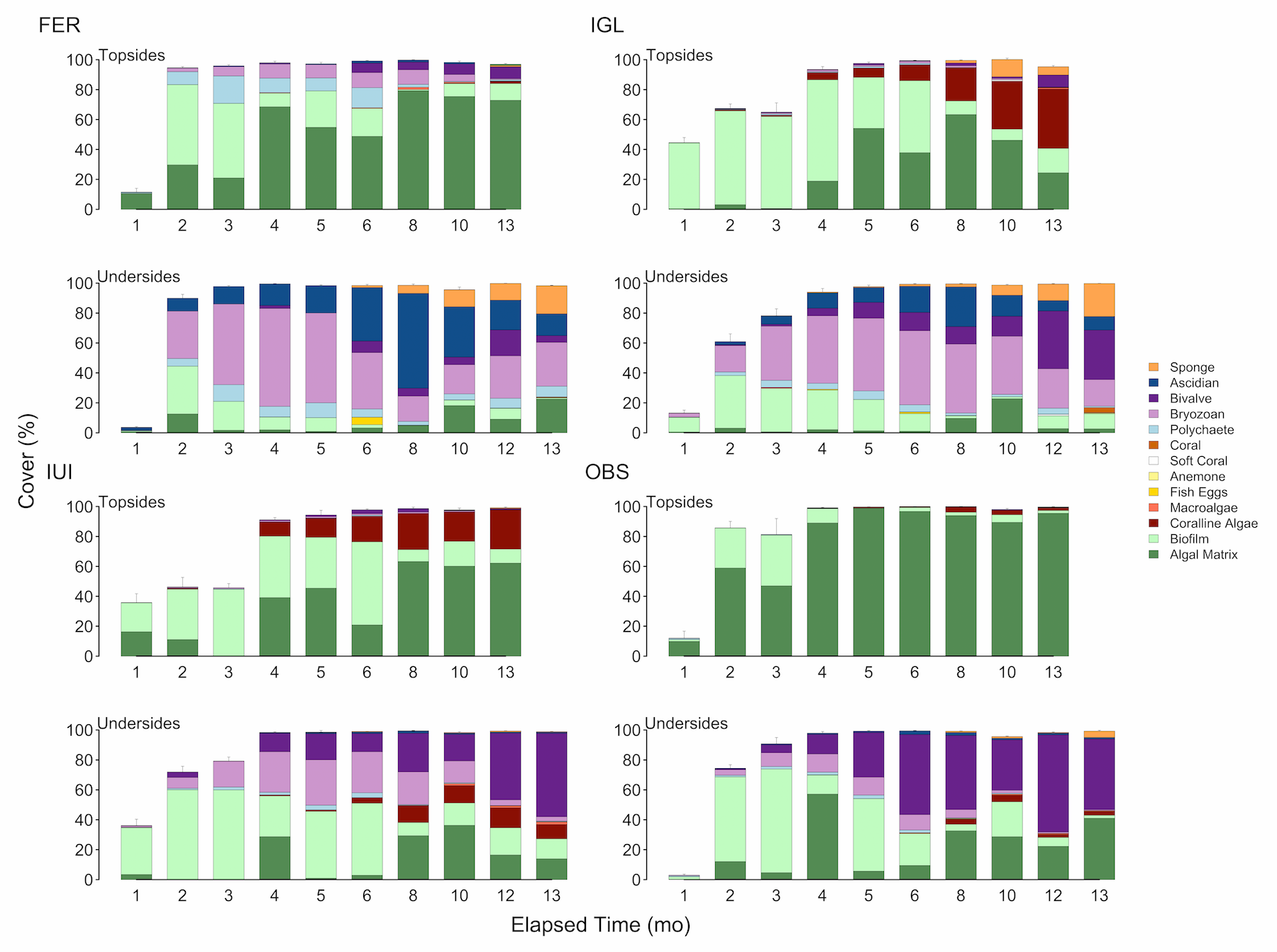

Supplement: S2 Fig — Change in planar cover (%) and composition of taxonomic groups on collector topsides and undersides at a suspended artificial reef (FER), a seafloor artificial reef (IGL), and 2 natural reefs (IUI, OBS) over 13 mo in the mensurative experiment (Oct 2015–Nov 2016). Bar heights are mean (+SE) of 10 arrays (2 collectors averaged per array) for artificial reefs and 5 arrays (4 collectors averaged per array) for natural reefs at each sampling interval. (TIF) [file pone.0212842.s002.tif]

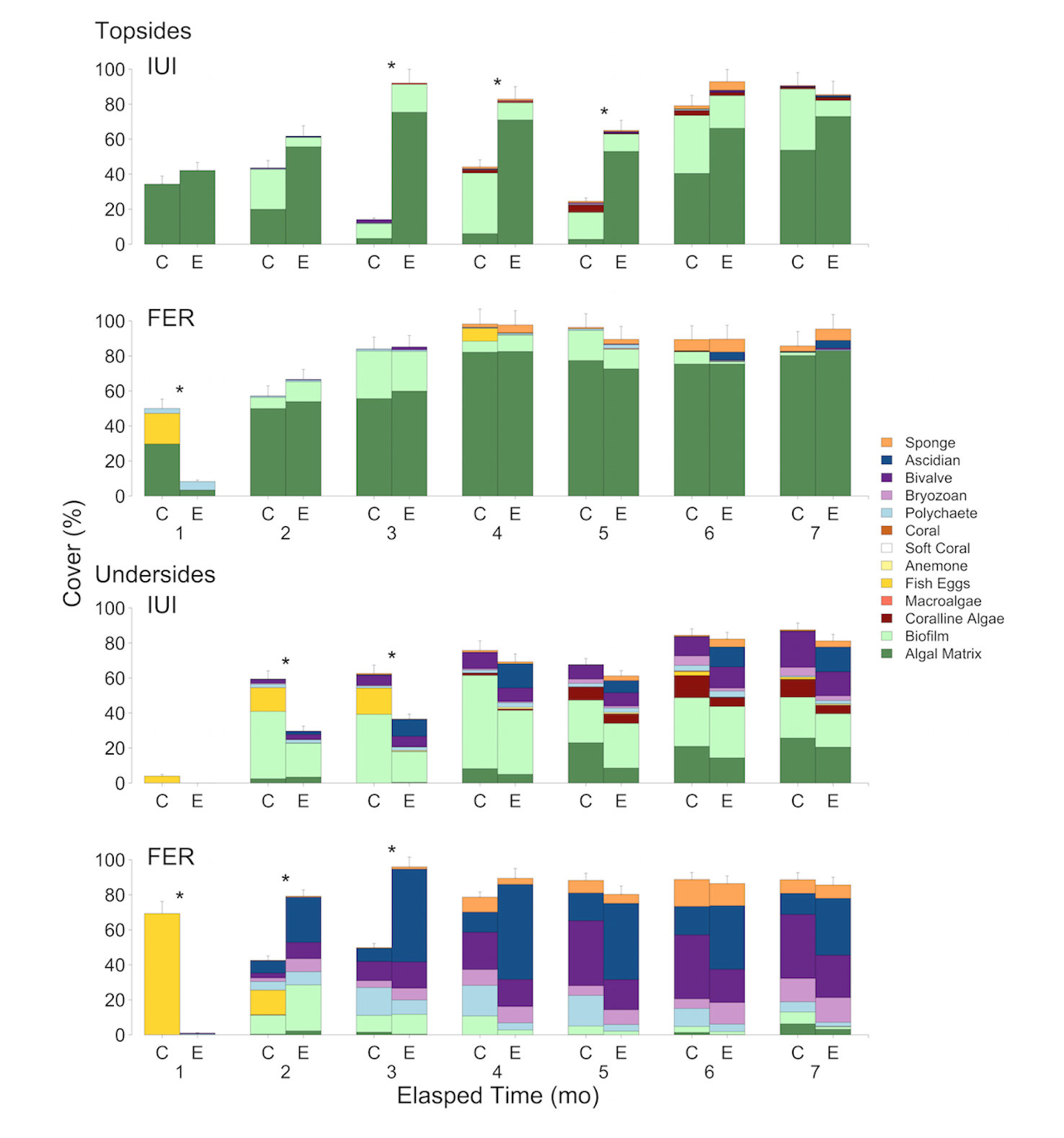

Supplement: S3 Fig — Change in planar cover (%) and composition of taxonomic groups on (A) collector topsides and (B) undersides for exclusion (E) and control (C) treatments at a suspended artificial reef (FER), and a natural reef (IUI) during the 7-mo manipulative experiment (April 2016–Nov 2016). Bar heights are mean (+SE) of 8 collectors for each treatment at each sampling interval. Asterisks indicate intervals when control and exclusion treatments are significantly different (α = 0.05). (TIF) [file pone.0212842.s003.tif]

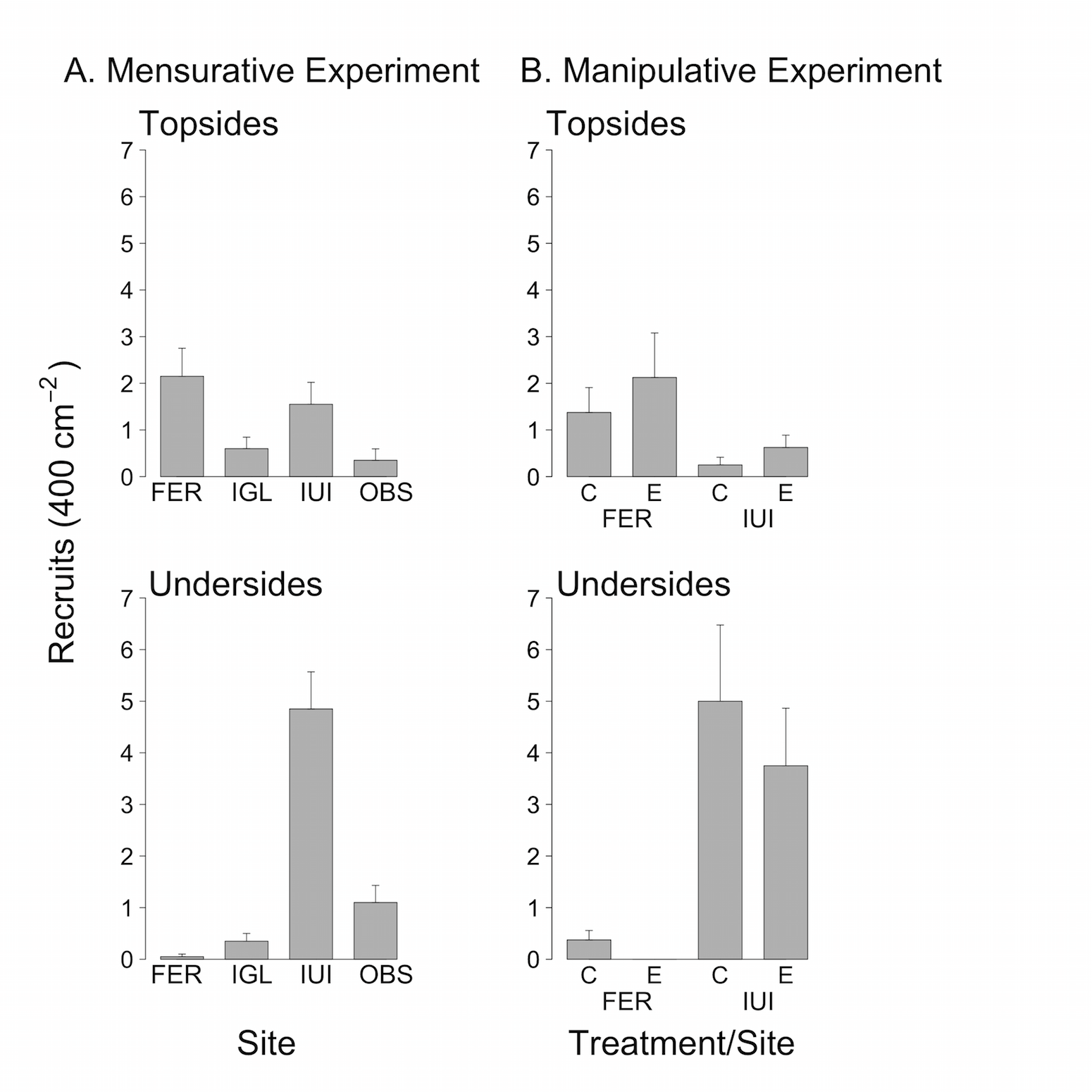

Supplement: S4 Fig — Mean (+SE) density of stony coral recruits (individuals 400 cm-2) on collector topsides and undersides for (A) two artificial reefs (FER, IGL; n = 10 arrays) and two natural reefs (IUI, OBS; n = 5 arrays) at the end of the mensurative experiment (Nov 2016), and for (B) control (C) and exclusion (E) treatments at one of the artificial reefs (FER) and one of the natural reefs (IUI) (n = 8 collectors for each site x treatment combination) at the end of the manipulative experiment (Nov 2016). (TIF) [file pone.0212842.s004.tif]
