# Supplementary material for: Benthic community succession on artificial and natural coral reefs in the northern Gulf of Aqaba, Red Sea
Source: PLoS One. 2019 Feb 27;14(2):e0212842. doi: 10.1371/journal.pone.0212842 (PMC6392313; doi:10.1371/journal.pone.0212842)
Supplement: S1 Table — Metadata from dives between 27 Mar and 19 Apr 2016 at a suspended artificial reef (FER), a seafloor artificial reef (IGL), and a natural reef (IUI). (DOCX) [file pone.0212842.s005.docx]

**S1 Table.**

|  |  |  |  |  |
| --- | --- | --- | --- | --- |
| Site | Date | Dive start | Dive end | Time analyzed |
| IUI | 29-Mar | 14:26 | 15:31 | 23.0 |
| IUI | 31-Mar | 9:33 | 10:36 | 48.3 |
| IUI | 01-Apr | 10:29 | 11:35 | 18.6 |
| IUI | 02-Apr | 11:30 | 12:30 | 45.8 |
| IUI | 07-Apr | 12:00 | 13:03 | 58.4 |
| IUI | 08-Apr | 10:25 | 11:30 | 45.6 |
| IUI | 08-Apr | 16:21 | 17:26 | 38.6 |
| IUI | 14-Apr | 11:28 | 12:38 | 43.3 |
|  |  |  | Total Time | 321.7 |
| FER | 11-Apr | 15:30 | 17:15 | 35.1 |
| FER | 12-Apr | 10:02 | 11:00 | 83.4 |
| FER | 13-Apr | 11:15 | 12:25 | 15.9 |
| FER | 13-Apr | 11:15 | 12:25 | 69.5 |
| FER | 14-Apr | 15:15 | 16:00 | 54.2 |
| FER | 19-Apr | 9:50 | 12:00 | 69.0 |
|  |  |  | Total Time | 327.1 |
| IGL | 09-Apr | 10:11 | 11:17 | 115.5 |
| IGL | 15-Apr | 10:25 | 11:30 | 121.8 |
|  |  |  | Total Time | 237.3 |
