# Supplementary material for: Benthic community succession on artificial and natural coral reefs in the northern Gulf of Aqaba, Red Sea
Source: PLoS One. 2019 Feb 27;14(2):e0212842. doi: 10.1371/journal.pone.0212842 (PMC6392313; doi:10.1371/journal.pone.0212842)
Supplement: S2 Table — Data are the lowest taxonomic level (Cl, Class; Or, Order; Fa, Family) identified for invertebrates on collector topsides (T) and undersides (U) at the end (Nov 2016) of a 13-mo mensurative experiment at a suspended artificial reef (FER), a seafloor artificial reef (IGL), and two natural reefs (IUI, OBS), and a 7-mo manipulative experiment at two of these sites (FER, IUI) for exclusion (E) and control (C) treatments. (DOCX) [file pone.0212842.s006.docx]

**S2 Table.**

| **Phylum/Class** | **Order/Family** | **Genus/Species** | **Mensurative** | **Manipulative** |
| --- | --- | --- | --- | --- |
| Porifera |  |  |  |  |
| Cl Calcarea | Or Clathrinida | |  |  |
|  | Fa Clathrinidae | *Clathrina coriacea* | IGL-U |  |
|  |  | *Clarintha* sp. | FER-U, IGL-U | IUI-UE |
|  | Or Leucosolenida | |  |  |
|  | Fa Grantiidae | *Grantia* sp. | FER-U, IGL-U | |
| Cl Demospongiae | Or Agelasida | |  |  |
|  | Fa Astroscleridae | *Astrosclera* sp. | FER-U, IGL-U | |
|  | Or Axinellida | |  |  |
|  | Fa Raspailiidae | *Ectyoplasia* sp. | FER-T/U, IGL-T/U, IUI-T/U, OBS-T/U | FER-TC/E, FER-UC/E, IUI-TC/E, IUI-UC/E |
|  | Or Bubarida |  |  |  |
|  | Fa Dictyonellidae | *Acanthella* sp. | FER-U, IGL-U | FER-UE |
|  | Or Chondrillida | |  |  |
|  | Fa Chondrillidae | *Chondrilla* sp. | FER-T, IGL-T | IUI-TC |
|  |  | sponge 13 | FER-U, IGL-U, OBS-U | FER-UC/E, IUI-UE |
|  |  | sponge 15 | FER-U, IUI-U | FER-UE, IUI-UC/E |
|  | Or Clionaida | |  |  |
|  | Fa Spirastrellidae | *Spirastrella coccinea* | IGL-U |  |
|  | Or Dictyoceratida | |  |  |
|  | Fa Thorectidae | *Hyrtios* sp. | IGL-U, OBS-U | |
|  | Or Hadromerida | |  |  |
|  | Fa Clionaidae | *Cliona* spp. | FER-U, IGL-T/U, IUI-T, OBS-T | FER-TC/E, FER-UC, IUI-TC/E, IUI-UE |
|  |  | *Pione* spp. | FER-T/U, IGL-T/U, IUI-T/U, OBS-T/U | FER-TE, FER- U (C, E), IUI-TC |
|  | Fa Tethyidae | *Tethya* spp. | FER-U, IGL-U, IUI-T/U | FER-UC/E, IUI-TE |
|  | Or Haplosclerida | |  |  |
|  | Fa Callyspongiidae | *Callyspongia siphonella* | FER-U, IGL-T/U, IUI-T/U, OBS-T/U | FER-TC/E, FER-UC/E, IUI-UC/E |
|  |  | *Callyspongia* spp. | FER-U, IGL-U | FER-UE, IUI-UC/E |
|  | Or Poecilosclerida | |  |  |
|  | Fa Crellidae | *Crella cyathophora* | IGL-U |  |
|  | Fa Desmacellidae | *Biemna* spp. | FER-U, IGL-U, IUI-U | FER-TE, FER-UC/E |
|  | Fa Hymedesmiidae | *Hemimycale arabica* | FER-U, IGL-T/U, IUI-U, OBS-T | FER-TC/E, FER-UC/E |
|  | Or Tetractinellida | |  |  |
|  | Fa Ancorinidae | sponge 5 | FER-U, IGL-U | FER-UC/E |
|  |  | sponge 6 | IGL-U, IUI-U | FER-UC |
|  |  | sponge 7 | IGL-T/U, IUI-U, OBS-U | FER-TE, FER-UC |
|  |  | sponge 23 | FER-U, IGL-U | FER-UE |
|  |  | sponge 24 | FER-U, IGL-U | FER-TE, FER-UC/E, IUI-TE |
|  |  | sponge 27 | IGL-U | IUI-UE |
|  |  |  |  |  |
| Cnidaria |  |  |  |  |
| Cl Anthozoa | Or Actiniaria | |  |  |
|  | Fa Actiniidae | *Anthopleura stellula* | FER-U, IGL-T/U | FER-UC |
|  |  | *Anthopleura* sp. | IGL-T/U |  |
|  | Fa Stichodactylidae | *Heteractis aurora* | IGL-U | IUI-UE |
|  | Or Alcyonacea | |  |  |
|  | Fa Alcyoniidae | *Rhytisma fulvum fulvum* | IGL-U, IUI-U, OBS-U | IUI-UC/E |
|  |  | soft coral 3b | FER-U |  |
|  | Fa Nephtheidae | *Dendronephthya* sp. | FER-U | IUI-TE |
|  | Fa Xeniidae | *Xenia* spp. | FER-U, IGL-U, OBS-U | IUI-UE |
|  | Or Scleractinia | |  |  |
|  | Fa Pocilloporidae | *Seriatopora sp.* | IGL-U |  |
|  |  | *Stylophora* spp. | FER-T/U, IGL-T/U, IUI-T/U, OBS-T/U | FER-TC/E, FER-UC, IUI-TE, IUI-UC/E |
|  | Fa Scleractinia incertae sedis | *Leptastrea* sp. | FER-T |  |
|  |  |  |  |  |
| Bryozoa |  |  |  |  |
| Cl Stenolaemata | Or Cyclostomata | bryozoan 14 | IGL-U | IUI-UC |
| Cl Gymnolaemata | Or Cheilostomata | |  |  |
|  | Fa Electridae | *Electra* spp. | FER-U, OBS-U | FER-UC, IUI-UC |
|  | Fa Candidae | *Licornia (Scrupocellaria)* spp. | FER-U | FER-UC/E |
|  | Fa Schizoporellidae | *Schizoporella errata* | FER-U, IUI-U | FER-UE, IUI-UC |
|  | Fa Smittinidae | *Parasmittina* spp. | FER-T/U, IGL-T/U, IUI-T/U, OBS-T/U | FER-UC/E, IUI-TC/E, IUI-UC/E |
|  | Unknown | bryozoan 8 | FER-T/U, IGL-T/U, OBS-U |  |
|  |  | bryozoan 9 |  | IUI-UC/E |
|  |  | bryozoan 10 | | IUI-UC |
|  |  | bryozoan 11 | | IUI-UE |
|  |  | bryozoan 15 | | IUI-UE |
|  |  |  |  |  |
| Mollusca |  |  |  |  |
| Cl Bivalvia | Or Arcoida |  |  |  |
|  | Fa Arcidae | *Barbatia* spp. | OBS-U |  |
|  | Or Ostreida |  |  |  |
|  | Fa Pteriidae | *Pteria aegyptiaca* | FER-U, IGL-U | FER-TE, FER-UC |
|  | Or Ostreoida | |  |  |
|  | Fa Ostreidae | *Alectryonella* spp. | FER-U, IGL-U | FER-UC |
|  |  | *Dendostrea* spp. | FER-T/U, IGL-T/U, IUI-U, OBS-U | FER-TE, FER-UC/E, IUI-UC |
|  |  | *Lopha* sp. | FER-U, IGL-U | FER-UC/E |
|  |  | *Ostrea* spp. | FER-T/U, IGL-T/U, IUI-U, OBS-T/U | FER-TE, FER-UC/E, IUI-TE, IUI-UC/E |
|  |  | *Saccostrea* spp. | FER-T/U, IGL-T/U | FER-TC/E, FER-UC/E, IUI-TC/E, IUI-UE |
|  |  | bivalve 2 | FER-U, IUI-T, OBS-T | FER-UC, IUI-TE |
|  |  | bivalve 20 | FER-T, IGL-U | FER-TE |
|  |  | bivalve 32 | IGL-U | IUI-TC, IUI-UC/E |
|  | Fa Mytilidae | *Modiolus* sp. | | IUI-UE |
|  | Or Pectinida |  |  |  |
|  | Fa Anomiidae | *Anomia* spp. | FER-T/U, IGL-U | FER-TE, FER- U (C, E) |
|  | Or Pectinoida | |  |  |
|  | Fa Spondylidae | *Spondylus* spp. | FER-U, IGL-U, IUI-U, OBS-U | IUI-UC |
|  |  | bivalve 33 |  | IUI-UE |
|  | Or Pterioida |  |  |  |
|  | Fa Pinnidae | *Pinna* sp. | FER-U, IGL-U | |
|  |  | *Streptopinna* spp. | FER-U | FER-UE |
|  | Fa Pinnoidea | bivalve 40 | IGL-U |  |
|  | Fa Pteriidae | *Pinctada* spp. | FER-T/U, IGL-U | IUI-TE, IUI-UE |
|  | Or Veneroida | |  |  |
|  | Fa Chamidae | *Chama* sp. | FER-U, IGL-U, OBS-U |  |
|  | Unknown | bivalve 15 |  | FER-UE |
| Cl Gastropoda | Or Littorinimorpha | |  |  |
|  | Fa Vermetidae | gastropod 4 | IGL-T, IUI-T/U, OBS-T/U | IUI-T |
|  |  | gastropod 12 | OBS-U | FER-U |
|  |  | gastropod 18 | IGL-T, OBS-T/U | |
|  |  | gastropod 22 | IGL-T |  |
|  |  |  |  |  |
| Annelida |  |  |  |  |
| Cl Polychaeta | Or Sabellida |  |  |  |
|  | Fa Serpulidae | *Hydroides* spp. | FER-T/U, IGL-U, IUI-T/U, OBS-U | FER-TE, FER-UC/E, IUI-TC, IUI-UC/E |
|  |  | *Josephella* sp. | IUI-U | IUI-UC |
|  |  | *Rhodopsis* sp. | IUI-U | IUI-UC |
|  |  | *Salmachina* sp. | IUI-U | IUI-UC |
|  |  | *Serpula* spp. | FER-T/U, IGL-T/U | IUI-TE, IUI-UC |
|  |  | *Spiraserpula* spp. | FER-U, IGL-U | |
|  |  | *Spirobranchus tetraceros* | FER-T/U, IGL-T/U, IUI-T/U, OBS-T/U | FER-TC/E, FER-UC/E, IUI-TC/E, IUI-UC/E |
|  |  | *Spirobranchus* spp. | IGL-U |  |
|  |  | *Spirorbis* spp. | FER-T/U, IGL-T/U, IUI-T/U, OBS-T/U | FER-TC/E, FER-UC/E, IUI-TC/E, IUI-UC/E |
|  |  | *Vermiliopsis* spp. | IGL-U |  |
|  | Unknown | polychaete 16 | FER-T/U, IGL-U | IUI-TE, IUI-UC/E |
|  |  | polychaete 19 | IGL-T |  |
|  |  |  |  |  |
| Chordata |  |  |  |  |
| Cl Ascidiacea | Or Aplousobranchia | |  |  |
|  | Fa Didemnidae | *Didemnum* spp. | FER-T/U, IGL-T/U, IUI-U, OBS-T/U | FER-TC/E, FER-UC/E, IUI-TC/E, IUI-UC/E |
|  |  | *Diplosoma simile* | IGL-T/U | IUI-TC/E, IUI-UE |
|  |  | *Diplosoma*sp. | FER-T/U, IGL-T/U | FER-UC/E, IUI-TE, IUI-UC/E |
|  | Or Enterogona | |  |  |
|  | Fa Ascidiidae | *Phallusia nigra* | FER-U | FER-TE, FER-UC/E |
|  | Fa Polyclinidae | *Aplidium* sp. | IGL-U | IUI-UE |
|  | Or Phlebobranchia | |  |  |
|  | Fa Corellidae | *Rhodosoma turcicum* | FER-U, IGL-U | FER-UE, IUI-UE |
|  | Or Pleurogona | |  |  |
|  | Fa Pyuridae | *Halocynthia spinosa* | FER-U, IGL-U | IUI-UE |
|  | Or Stolidobranchia | |  |  |
|  | Fa Styelidae | *Eusynstyela latericius* morph 2 | IGL-U, IUI-U, OBS-U | IUI-UC |
|  |  | *Eusynstyela latericius* morph 1 | FER-U, IGL-U, IUI-U, OBS-U | IUI-UE |
|  |  | *Botrylloides* spp. | FER-T/U, IGL-U | FER-TE, FER-UC/E |
|  |  | *Polycarpa mytiligera* | FER-U, IGL-T/U, OBS-U | FER-UE, IUI-UC/E |
|  |  | *Polycarpa* spp. | FER-U, IGL-T/U | IUI-UE |
|  |  | *Styela canopus* | IGL-U |  |
|  | Fa Pyuridae | *Herdmania momus* | FER-U | FER-TE, FER-UC/E |
|  | Unknown |  |  |  |
|  |  | ascidian 11 |  | FER-UC/E |
|  |  | ascidian 12 | IGL-U, OBS-U | |
|  |  | ascidian 14 |  | FER-UE |
|  |  | ascidian 17 | OBS-U | FER-UE |
|  |  | ascidian 19 | IGL-U, IUI-U | |
|  |  | ascidian 25 |  | IUI-UE |
|  |  | ascidian 27 |  | IUI-UE |
|  |  | ascidian 32 |  | IUI-UE |
|  |  | ascidian 33 | IGL-U | IUI-UE |
|  |  | ascidian 37 | FER-U | IUI-TE |
|  |  | ascidian 38 | IGL-U |  |
|  |  | ascidian 39 | IGL-U |  |
|  |  | ascidian 40 | IGL-U |  |
|  |  | ascidian 41 | IGL-U |  |
|  |  | ascidian 42 | IGL-U |  |
|  |  | ascidian 44 | IGL-U |  |

^a^Identification References and Experts:

*Anemones:* Vine P. *Red Sea Invertebrates*. IMMEL Publishing: London; 1996.

*Ascidians:* Noa Shenkar (Tel-Aviv University), Gil Kopolovitz (IUI)

*Bivalves:*

Edelman-Furstenberg Y, Faershtein G. Molluscan fauna of the Gulf of Elat: indicators of ecological change. Geological Survey of Israël; 2010.

Vine P. *Red Sea Invertebrates*. IMMEL Publishing: London; 1996.

Wronski T. The molluscan bio-fouling community on the Red Sea pearl oyster beds: (Mollusca: Pteriidae). Zoology in the Middle East. 2010 Jan 1;51(1):67-73.

Zuschin M, Hohenegger J, Steininger FF. A comparison of living and dead molluscs on coral reef associated hard substrata in the northern Red Sea—implications for the fossil record. Palaeogeography, Palaeoclimatology, Palaeoecology. 2000 Jun 1;159(1-2):167-90.

Zuschin M, Hohenegger J, Steininger F. Molluscan assemblages on coral reefs and associated hard substrata in the northern Red Sea. Coral Reefs. 2001 Sep 1;20(2):107-16.

*Bryozoans;* Noga Sokolover (Tel-Aviv University)

*Polychaetes:* Harry ten Hove (Naturalis Biodiversity Centre, Netherlands)

*Stony corals:* Tom Shlesinger (Tel-Aviv University); Dor Shefy (Ben-Gurion University of the Negev)

V

ine P. *Red Sea Invertebrates*. IMMEL Publishing: London; 1996.

*Sponges:*

Erpenbeck D, Voigt O, Al-Aidaroos AM, Berumen ML, Büttner G, Catania D, Guirguis AN, Paulay G, Schätzle S, Wörheide G. Molecular biodiversity of Red Sea demosponges. Marine pollution bulletin. 2016 Apr 30;105(2):507-14.

Hooper JN. Sponguide: guide to sponge collection and identification. Queensland Museum; 2000.

Ilan M, Gugel J, Van Soest R. Taxonomy, reproduction and ecology of new and known Red Sea sponges. Sarsia: North Atlantic Marine Science. 2004 Dec 1;89(6):388-410.

Richter C, Wunsch M, Rasheed M, KoÈtter I, Badran MI. Endoscopic exploration of Red Sea coral reefs reveals dense populations of cavity-dwelling sponges. Nature. 2001 Oct;413(6857):726.

Vine P. *Red Sea Invertebrates*. IMMEL Publishing: London; 1996.

World Register of Marine Species: World Porifera Database (http://www.marinespecies.org/porifera/)
