# Supplementary material for: Benthic community succession on artificial and natural coral reefs in the northern Gulf of Aqaba, Red Sea
Source: PLoS One. 2019 Feb 27;14(2):e0212842. doi: 10.1371/journal.pone.0212842 (PMC6392313; doi:10.1371/journal.pone.0212842)
Supplement: S4 Table — Analysis indicates the contribution of different taxonomic groups to dissimilarity in the composition of planar cover (%) between combinations of sites (FER, IGL, IUI, OBS), for topsides and undersides of collectors, at the end of the 13-mo experiment. (DOCX) [file pone.0212842.s008.docx]

**S4 Table.**

| *Topsides* |  |  |  |  |  |  |
| --- | --- | --- | --- | --- | --- | --- |
| Group | Average Abundance | Average Abundance | Average Dissimilarity | Dissimilarity/SD | Contributing % | Cumulative % |
|  | Group FER | Group IGL |  |  |  |  |
| Coralline algae | 0.1 | 0.7 | 14.2 | 3.0 | 32.9 | 32.9 |
| Algal matrix | 1.0 | 0.5 | 13.0 | 2.3 | 30.1 | 63.0 |
| Sponge | 0.1 | 0.2 | 3.7 | 1.6 | 8.6 | 71.7 |
|  | Group FER | Group IUI |  |  |  |  |
| Coralline algae | 0.1 | 0.5 | 11.1 | 2.8 | 34.4 | 34.4 |
| Algal matrix | 1.0 | 0.9 | 5.6 | 1.3 | 17.4 | 51.8 |
| Bivalve | 0.3 | 0.1 | 5.3 | 1.7 | 16.3 | 68.0 |
| Biofilm | 0.3 | 0.3 | 3.4 | 1.5 | 10.6 | 78.7 |
|  | Group FER | Group OBS |  |  |  |  |
| Algal matrix | 1.0 | 1.4 | 9.1 | 2.2 | 26.9 | 26.9 |
| Bivalve | 0.3 | 0.0 | 7.0 | 2.4 | 20.7 | 47.6 |
| Biofilm | 0.3 | 0.1 | 7.0 | 2.0 | 20.6 | 68.2 |
| Coralline algae | 0.1 | 0.1 | 2.6 | 1.4 | 7.7 | 75.9 |
|  | Group IUI | Group OBS |  |  |  |  |
| Algal matrix | 0.9 | 1.4 | 12.7 | 1.9 | 34.8 | 34.8 |
| Coralline algae | 0.5 | 0.1 | 11.4 | 2.8 | 31.3 | 66.1 |
| Biofilm | 0.3 | 0.1 | 6.4 | 1.7 | 17.4 | 83.6 |
|  | Group IGL | Group IUI |  |  |  |  |
| Algal matrix | 0.5 | 0.9 | 11.2 | 1.7 | 34.0 | 34.0 |
| Coralline algae | 0.7 | 0.5 | 5.4 | 1.3 | 16.2 | 50.2 |
| Bivalve | 0.3 | 0.1 | 5.1 | 1.5 | 15.4 | 65.6 |
| Biofilm | 0.4 | 0.3 | 4.1 | 1.4 | 12.5 | 78.1 |
|  | Group IGL | Group OBS |  |  |  |  |
| Algal matrix | 0.5 | 1.4 | 22.9 | 3.7 | 36.6 | 36.6 |
| Coralline algae | 0.7 | 0.1 | 14.7 | 2.9 | 23.5 | 60.2 |
| Biofilm | 0.4 | 0.1 | 8.6 | 2.3 | 13.7 | 73.9 |
|  |  |  |  |  |  |  |
| Undersides |  |  |  |  |  |  |
| Group | Average Abundance | Average Abundance | Average Dissimilarity | Dissimilarity/SD | Contributing % | Cumulative % |
|  | Group FER | Group IGL |  |  |  |  |
| Bivalve | 0.2 | 0.6 | 8.9 | 2.5 | 22.8 | 22.8 |
| Algal matrix | 0.5 | 0.1 | 7.2 | 2.6 | 18.5 | 41.4 |
| Biofilm | 0.1 | 0.3 | 5.3 | 2.4 | 13.8 | 55.1 |
| Bryozoan | 0.6 | 0.4 | 4.7 | 1.2 | 12.2 | 67.3 |
| Polychaete | 0.3 | 0.1 | 3.9 | 1.5 | 10.0 | 77.3 |
|  | Group FER | Group IUI |  |  |  |  |
| Bivalve | 0.2 | 0.9 | 14.2 | 3.1 | 23.0 | 23.0 |
| Bryozoan | 0.6 | 0.2 | 8.7 | 1.9 | 14.2 | 37.2 |
| Sponge | 0.4 | 0.1 | 8.3 | 5.7 | 13.5 | 50.6 |
| Ascidian | 0.4 | 0.1 | 6.8 | 2.7 | 11.0 | 61.7 |
| Biofilm | 0.1 | 0.4 | 6.4 | 2.4 | 10.4 | 72.0 |
|  | Group FER | Group OBS |  |  |  |  |
| Bivalve | 0.2 | 0.8 | 12.6 | 3.3 | 24.1 | 24.1 |
| Bryozoan | 0.6 | 0.1 | 10.8 | 2.4 | 20.7 | 44.8 |
| Ascidian | 0.4 | 0.1 | 6.4 | 2.5 | 12.2 | 57.0 |
| Sponge | 0.4 | 0.2 | 5.3 | 3.7 | 10.0 | 67.0 |
| Algal matrix | 0.5 | 0.7 | 5.0 | 1.6 | 9.6 | 76.7 |
|  | Group IUI | Group OBS |  |  |  |  |
| Algal matrix | 0.4 | 0.7 | 7.1 | 1.9 | 23.6 | 23.6 |
| Biofilm | 0.4 | 0.1 | 5.0 | 2.0 | 16.6 | 40.2 |
| Bivalve | 0.9 | 0.8 | 3.8 | 1.2 | 12.6 | 52.8 |
| Sponge | 0.1 | 0.2 | 3.4 | 4.2 | 11.3 | 64.1 |
| Coralline algae | 0.3 | 0.2 | 3.3 | 1.5 | 11.1 | 75.2 |
|  | Group IGL | Group IUI |  |  |  |  |
| Sponge | 0.5 | 0.1 | 9.9 | 3.2 | 19.5 | 19.5 |
| Coralline algae | 0.0 | 0.3 | 6.2 | 3.5 | 13.6 | 33.1 |
| Bryozoan | 0.4 | 0.2 | 5.7 | 1.7 | 12.5 | 45.6 |
| Bivalve | 0.6 | 0.9 | 5.4 | 1.5 | 11.8 | 57.4 |
| Algal matrix | 0.1 | 0.4 | 5.1 | 1.8 | 11.1 | 68.5 |
| Ascidian | 0.3 | 0.1 | 5.0 | 2.3 | 11.0 | 79.5 |
|  | Group IGL | Group OBS |  |  |  |  |
| Algal matrix | 0.1 | 0.7 | 12.1 | 3.3 | 25.9 | 25.9 |
| Bryozoan | 0.4 | 0.1 | 7.3 | 2.0 | 15.6 | 41.5 |
| Sponge | 0.5 | 0.2 | 5.9 | 2.1 | 12.7 | 54.2 |
| Ascidian | 0.3 | 0.1 | 4.6 | 2.1 | 9.8 | 64.0 |
| Bivalve | 0.6 | 0.8 | 4.0 | 1.5 | 8.5 | 72.5 |
