# Supplementary material for: Benthic community succession on artificial and natural coral reefs in the northern Gulf of Aqaba, Red Sea
Source: PLoS One. 2019 Feb 27;14(2):e0212842. doi: 10.1371/journal.pone.0212842 (PMC6392313; doi:10.1371/journal.pone.0212842)
Supplement: S5 Table — Analysis examines the effect of site (fixed factor: FER, IGL, IUI, OBS) on the composition of invertebrate biomass (g 400 cm-2) on topsides and undersides of collectors at the end of the 13-mo experiment. Also shown are pairwise comparisons using the PERMANOVA t-statistic. Tests are based on 999 permutations. Significant results in bold. (DOCX) [file pone.0212842.s009.docx]

**S5 Table.**

| Source | *df* | MS | *Pseudo-F* | *p(perm)* |
| --- | --- | --- | --- | --- |
| *Topsides* |  |  |  |  |
| Site | 3 | 3739.5 | 13.9 | **0.001** |
| Residual | 26 | 268.3 |  |  |
|  |  |  |  |  |
| *Undersides* |  |  |  |  |
| Site | 3 | 1338.5 | 13.4 | **0.001** |
| Residual | 26 | 100.1 |  |  |
|  |  |  |  |  |
|  |  | *t* |  | *p(perm)* |
| *Topsides* |  |  |  |  |
| FER vs. IGL |  | 3.7 |  | **0.001** |
| FER vs. IUI |  | 4.0 |  | **0.002** |
| FER vs. OBS |  | 3.2 |  | **0.001** |
| IGL vs. IUI |  | 4.1 |  | **0.001** |
| IGL vs. OBS |  | 4.2 |  | **0.001** |
| IUI vs. OBS |  | 3.2 |  | **0.009** |
|  |  |  |  |  |
| *Undersides* |  |  |  |  |
| FER vs. IGL |  | 2.1 |  | **0.004** |
| FER vs. IUI |  | 4.0 |  | **0.003** |
| FER vs. OBS |  | 4.5 |  | **0.001** |
| IGL vs. IUI |  | 4.1 |  | **0.002** |
| GL vs. OBS |  | 4.0 |  | **0.001** |
| IUI vs. OBS |  | 2.6 |  | **0.009** |
