# Supplementary material for: Benthic community succession on artificial and natural coral reefs in the northern Gulf of Aqaba, Red Sea
Source: PLoS One. 2019 Feb 27;14(2):e0212842. doi: 10.1371/journal.pone.0212842 (PMC6392313; doi:10.1371/journal.pone.0212842)
Supplement: S6 Table — Analysis indicates the contribution of different taxonomic groups to dissimilarity in the composition of invertebrate biomass (g 400 cm-2) between combinations of sites (FER, IGL, IUI, OBS), for topsides and undersides of collectors, at the end of the 13-mo experiment. (DOCX) [file pone.0212842.s010.docx]

**S6 Table.**

| *Topsides* |  |  |  |  |  |  |
| --- | --- | --- | --- | --- | --- | --- |
| Group | Average Abundance | Average Abundance | Average Dissimilarity | Dissimilarity/SD | Contributing % | Cumulative % |
|  | Group FER | Group IGL |  |  |  |  |
| Sponge | 0.2 | 1.2 | 14.7 | 2.4 | 36.6 | 36.6 |
| Bivalve | 1.2 | 0.9 | 7.2 | 1.5 | 17.8 | 54.5 |
| Coral | 0.6 | 0.2 | 5.8 | 1.7 | 14.3 | 68.8 |
| Ascidian | 0.2 | 0.4 | 4.8 | 1.5 | 12.0 | 80.8 |
|  | Group FER | Group IUI |  |  |  |  |
| Bivalve | 1.2 | 0.2 | 20.3 | 2.3 | 36.6 | 36.6 |
| Polychaete | 0.8 | 0.2 | 11.3 | 2.5 | 20.3 | 56.9 |
| Sponge | 0.2 | 0.7 | 10.5 | 2.0 | 18.9 | 75.8 |
|  | Group FER | Group OBS |  |  |  |  |
| Bivalve | 1.2 | 0.4 | 21.1 | 1.7 | 35.6 | 35.6 |
| Coral | 0.6 | 0.1 | 11.1 | 1.9 | 18.6 | 54.2 |
| Bryozoan | 0.4 | 0.0 | 9.1 | 1.7 | 15.3 | 69.5 |
| Polychaete | 0.8 | 0.6 | 9.1 | 1.3 | 15.3 | 84.9 |
|  | Group IUI | Group OBS |  |  |  |  |
| Sponge | 0.7 | 0.0 | 22.5 | 3.1 | 29.5 | 29.5 |
| Polychaete | 0.2 | 0.6 | 16.0 | 1.6 | 21.0 | 50.5 |
| Bryozoan | 0.4 | 0.0 | 13.6 | 1.6 | 17.8 | 68.2 |
| Coral | 0.4 | 0.1 | 12.0 | 1.6 | 15.7 | 84.0 |
|  | Group IGL | Group IUI |  |  |  |  |
| Bivalve | 0.9 | 0.2 | 13.7 | 2.7 | 28.2 | 28.2 |
| Sponge | 1.2 | 0.7 | 9.0 | 1.5 | 18.7 | 46.9 |
| Polychaete | 0.7 | 0.2 | 8.5 | 1.4 | 17.6 | 64.5 |
| Ascidian | 0.4 | 0.0 | 7.1 | 1.8 | 14.8 | 79.3 |
|  | Group IGL | Group OBS |  |  |  |  |
| Sponge | 1.2 | 0.0 | 24.2 | 3.2 | 38.7 | 38.7 |
| Bivalve | 0.9 | 0.4 | 12.6 | 1.5 | 20.1 | 58.8 |
| Polychaete | 0.7 | 0.6 | 8.5 | 1.2 | 13.5 | 72.3 |
| *Undersides* |  |  |  |  |  |  |
| Group | Average Abundance | Average Abundance | Average Dissimilarity | Dissimilarity/SD | Contributing % | Cumulative % |
|  | Group FER | Group IGL |  |  |  |  |
| Bivalve | 2.0 | 2.4 | 4.0 | 1.6 | 21.2 | 21.2 |
| Ascidian | 1.6 | 1.4 | 3.4 | 1.5 | 18.0 | 39.2 |
| Bryozoan | 1.4 | 1.1 | 2.8 | 1.2 | 14.8 | 54.0 |
| Sponge | 1.1 | 0.9 | 2.2 | 1.5 | 12.0 | 66.0 |
| Soft coral | 0.1 | 0.3 | 1.9 | 1.5 | 9.9 | 75.9 |
|  | Group FER | Group IUI |  |  |  |  |
| Ascidian | 1.6 | 0.5 | 8.8 | 2.3 | 27.8 | 27.8 |
| Bivalve | 2.0 | 1.7 | 5.2 | 1.4 | 16.5 | 44.4 |
| Coral | 0.0 | 0.6 | 5.0 | 10.3 | 15.7 | 60.0 |
| Bryozoan | 1.4 | 0.9 | 4.7 | 1.5 | 14.9 | 75.0 |
|  | Group FER | Group OBS |  |  |  |  |
| Ascidian | 1.6 | 0.8 | 6.9 | 2.1 | 21.7 | 21.7 |
| Bryozoan | 1.4 | 0.6 | 6.6 | 2.3 | 20.8 | 42.4 |
| Sponge | 1.1 | 0.3 | 6.2 | 2.3 | 19.3 | 61.8 |
| Bivalve | 2.0 | 2.4 | 4.6 | 1.7 | 14.3 | 76.1 |
|  | Group IUI | Group OBS |  |  |  |  |
| Bivalve | 1.7 | 2.4 | 7.1 | 1.6 | 33.2 | 33.2 |
| Ascidian | 0.5 | 0.8 | 3.0 | 2.1 | 14.1 | 47.3 |
| Polychaete | 0.8 | 0.7 | 2.6 | 1.6 | 12.3 | 59.6 |
| Bryozoan | 0.9 | 0.6 | 2.6 | 1.3 | 12.3 | 71.8 |
|  | Group IGL | Group IUI |  |  |  |  |
| Ascidian | 1.4 | 0.5 | 7.0 | 2.2 | 23.9 | 23.9 |
| Bivalve | 2.4 | 1.7 | 6.0 | 1.6 | 20.6 | 44.5 |
| Polychaete | 1.2 | 0.8 | 3.6 | 3.9 | 12.2 | 56.7 |
| Coral | 0.2 | 0.6 | 3.4 | 1.8 | 11.6 | 68.3 |
| Sponge | 0.9 | 0.6 | 3.1 | 2.3 | 10.8 | 79.0 |
|  | Group IGL | Group OBS |  |  |  |  |
| Ascidian | 1.4 | 0.8 | 5.0 | 1.7 | 19.1 | 19.1 |
| Sponge | 0.9 | 0.3 | 4.9 | 2.2 | 18.6 | 37.7 |
| Polychaete | 1.2 | 0.7 | 4.2 | 1.8 | 16.1 | 53.8 |
| Bryozoan | 1.1 | 0.6 | 3.8 | 2.1 | 14.7 | 68.5 |
| Coral | 0.2 | 0.4 | 2.4 | 2.1 | 9.2 | 77.7 |
