# Supplementary material for: Benthic community succession on artificial and natural coral reefs in the northern Gulf of Aqaba, Red Sea
Source: PLoS One. 2019 Feb 27;14(2):e0212842. doi: 10.1371/journal.pone.0212842 (PMC6392313; doi:10.1371/journal.pone.0212842)
Supplement: S7 Table — Analysis examines the effects of site (FER, IUI) and treatment (exclusion, control) on the composition of planar cover (%) on topsides and undersides of collectors at the end of the 7-mo experiment. Significant results in bold. (DOCX) [file pone.0212842.s011.docx]

### **S7 Table.**

|  | *df* | MS | *Pseudo-F* | *p(perm)* |
| --- | --- | --- | --- | --- |
| *Topsides* |  |  |  |  |
| Site | 1 | 2645.4 | 13.4 | **0.001** |
| Treatment | 1 | 551.2 | 2.8 | 0.076 |
| Site x Treatment | 1 | 343.2 | 1.7 | 0.150 |
| Residual | 28 | 197.9 |  |  |
|  |  |  |  |  |
| *Undersides* |  |  |  |  |
| Site | 1 | 6278.4 | 37.4 | **0.001** |
| Treatment | 1 | 1218.1 | 7.3 | **0.001** |
| Site x Treatment | 1 | 62.2 | 0.4 | 0.823 |
| Residual | 28 | 167.9 |  |  |
