# Supplementary material for: Benthic community succession on artificial and natural coral reefs in the northern Gulf of Aqaba, Red Sea
Source: PLoS One. 2019 Feb 27;14(2):e0212842. doi: 10.1371/journal.pone.0212842 (PMC6392313; doi:10.1371/journal.pone.0212842)
Supplement: S8 Table — Analysis indicates the contribution of different taxonomic groups to dissimilarity of the composition of planar cover (%) between treatments (exclusion, E; control, C) at an artificial (FER) and natural (IUI) reef, and between reefs for each treatment, on topsides and undersides of collectors at the end of the 7-mo experiment. (DOCX) [file pone.0212842.s012.docx]

**S8 Table.**

| *Topsides* |  |  |  |  |  |  |
| --- | --- | --- | --- | --- | --- | --- |
| Group | Average Abundance | Average Abundance | Average Dissimilarity | Dissimilarity/SD | Contributing % | Cumulative % |
| *FER* | Group E | Group C |  |  |  |  |
| Algal Matrix | 1.2 | 1.1 | 8.7 | 0.9 | 33.0 | 33.0 |
| Sponges | 0.2 | 0.1 | 5.5 | 1.1 | 20.6 | 53.6 |
| Ascidians | 0.2 | 0.0 | 5.0 | 1.0 | 19.0 | 72.6 |
|  |  |  |  |  |  |  |
| *IUI* | Group E | Group C |  |  |  |  |
| Algal Matrix | 1.0 | 0.8 | 14.5 | 1.2 | 40.3 | 40.3 |
| Biofilm | 0.3 | 0.6 | 12.7 | 1.5 | 35.2 | 75.5 |
|  |  |  |  |  |  |  |
| *Exclusion* | Group FER | Group IUI |  |  |  |  |
| Algal Matrix | 1.2 | 1.0 | 9.9 | 0.8 | 27.3 | 27.3 |
| Biofilm | 0.0 | 0.3 | 9.0 | 2.3 | 24.7 | 52.0 |
| Sponges | 0.2 | 0.0 | 5.7 | 1.0 | 15.6 | 67.6 |
| Ascidians | 0.2 | 0.0 | 4.9 | 1.0 | 13.5 | 81.0 |
|  |  |  |  |  |  |  |
| *Control* | Group FER | Group IUI |  |  |  |  |
| Biofilm | 0.1 | 0.6 | 18.0 | 1.7 | 41.1 | 41.1 |
| Algal Matrix | 1.1 | 0.8 | 15.6 | 1.3 | 35.8 | 76.9 |
|  |  |  |  |  |  |  |
| *Undersides* |  |  |  |  |  |  |
| Group | Average Abundance | Average Abundance | Average Dissimilarity | Dissimilarity/SD | Contributing % | Cumulative % |
| *FER* | Group E | Group C |  |  |  |  |
| Ascidians | 0.6 | 0.3 | 7.5 | 1.4 | 25.8 | 25.8 |
| Biofilm | 0.1 | 0.2 | 4.5 | 1.4 | 15.3 | 41.1 |
| Bivalves | 0.5 | 0.7 | 4.5 | 1.4 | 15.2 | 56.3 |
| Bryozoans | 0.4 | 0.4 | 3.4 | 1.3 | 11.6 | 67.9 |
| Algal Matrix | 0.2 | 0.2 | 3.4 | 1.5 | 11.5 | 79.4 |
|  |  |  |  |  |  |  |
| *IUI* | Group E | Group C |  |  |  |  |
| Ascidians | 0.3 | 0.0 | 6.3 | 0.9 | 21.6 | 21.6 |
| Coralline Algae | 0.2 | 0.3 | 3.7 | 1.4 | 12.6 | 34.2 |
| Sponges | 0.2 | 0.0 | 3.3 | 1.2 | 11.2 | 45.3 |
| Algal Matrix | 0.5 | 0.5 | 3.2 | 1.3 | 11.0 | 56.4 |
| Bryozoans | 0.1 | 0.2 | 3.1 | 1.4 | 10.7 | 67.0 |
| Biofilm | 0.4 | 0.5 | 2.8 | 1.5 | 9.6 | 76.6 |
|  |  |  |  |  |  |  |
| *Exclusion* | Group FER | Group IUI |  |  |  |  |
| Ascidians | 0.6 | 0.3 | 9.7 | 1.7 | 20.9 | 20.9 |
| Biofilm | 0.1 | 0.4 | 8.8 | 2.3 | 19.1 | 40.0 |
| Algal Matrix | 0.2 | 0.5 | 7.0 | 2.0 | 15.1 | 55.0 |
| Bryozoans | 0.4 | 0.1 | 5.6 | 1.9 | 12.0 | 67.1 |
| Coralline Algae | 0.0 | 0.2 | 4.6 | 1.7 | 10.0 | 77.0 |
|  |  |  |  |  |  |  |
| *Control* | Group FER | Group IUI |  |  |  |  |
| Algal Matrix | 0.2 | 0.5 | 7.2 | 1.8 | 15.0 | 15.0 |
| Coralline Algae | 0.0 | 0.3 | 7.1 | 2.6 | 14.8 | 29.8 |
| Ascidians | 0.3 | 0.0 | 6.7 | 1.6 | 13.9 | 43.7 |
| Biofilm | 0.2 | 0.5 | 6.6 | 1.7 | 13.7 | 57.4 |
| Sponges | 0.3 | 0.0 | 5.6 | 2.2 | 11.6 | 69.0 |
| Bryozoans | 0.4 | 0.2 | 4.8 | 1.4 | 10.1 | 79.1 |
