# Supplementary material for: Benthic community succession on artificial and natural coral reefs in the northern Gulf of Aqaba, Red Sea
Source: PLoS One. 2019 Feb 27;14(2):e0212842. doi: 10.1371/journal.pone.0212842 (PMC6392313; doi:10.1371/journal.pone.0212842)
Supplement: S9 Table — Analysis examines the effects of site (FER, IUI) and treatment (exclusion, control) on the composition of invertebrate biomass (g 400 cm-2) on topsides and undersides of collectors at the end of the 7-mo experiment. Significant results in bold. (DOCX) [file pone.0212842.s013.docx]

### **S9 Table.**

|  | *df* | MS | *Pseudo-F* | *p(perm)* |
| --- | --- | --- | --- | --- |
| *Topsides* |  |  |  |  |
| Site | 1 | 5802.1 | 9.3 | **0.001** |
| Treatment | 1 | 7385.2 | 11.9 | **0.001** |
| Site x Treatment | 1 | 1344.0 | 2.2 | 0.094 |
| Residual | 28 | 622.4 |  |  |
|  |  |  |  |  |
| *Undersides* |  |  |  |  |
| Site | 1 | 8987.0 | 33.6 | **0.001** |
| Treatment | 1 | 3068.0 | 11.5 | **0.001** |
| Site x Treatment | 1 | 630.7 | 2.4 | **0.048** |
| Residual | 28 | 267.3 |  |  |
