# Supplementary material for: Benthic community succession on artificial and natural coral reefs in the northern Gulf of Aqaba, Red Sea
Source: PLoS One. 2019 Feb 27;14(2):e0212842. doi: 10.1371/journal.pone.0212842 (PMC6392313; doi:10.1371/journal.pone.0212842)
Supplement: S10 Table — Analysis indicates the contribution of different taxonomic groups to dissimilarity of the composition of invertebrate biomass (g 400 cm-2) between treatments (exclusion, E; control, C) at an artificial (FER) and natural (IUI) reef, and between reefs for each treatment, on topsides and undersides of collectors at the end of the 7-mo experiment. (DOCX) [file pone.0212842.s014.docx]

**S10 Table.**

| *Topsides* |  |  |  |  |  |  |
| --- | --- | --- | --- | --- | --- | --- |
| Group | Average Abundance | Average Abundance | Average Dissimilarity | Dissimilarity/SD | Contributing % | Cumulative % |
| *FER* | Group E | Group C |  |  |  |  |
| Bivalves | 1.5 | 0.1 | 30.9 | 2.7 | 45.8 | 45.8 |
| Ascidians | 0.8 | 0.1 | 13.7 | 1.4 | 20.2 | 66.0 |
| Sponges | 0.3 | 0.5 | 10.9 | 0.9 | 16.1 | 82.1 |
|  |  |  |  |  |  |  |
| *IUI* | Group E | Group C |  |  |  |  |
| Sponges | 0.3 | 0.6 | 25.0 | 1.0 | 31.8 | 31.8 |
| Ascidians | 0.5 | 0.0 | 17.7 | 1.1 | 22.5 | 54.4 |
| Bivalves | 0.4 | 0.1 | 11.6 | 0.9 | 14.7 | 69.1 |
| Polychaetes | 0.2 | 0.2 | 9.2 | 0.9 | 11.7 | 80.8 |
|  |  |  |  |  |  |  |
| *Exclusion* | Group FER | Group IUI |  |  |  |  |
| Bivalves | 1.5 | 0.4 | 26.2 | 1.4 | 40.0 | 40.0 |
| Ascidians | 0.8 | 0.5 | 12.9 | 1.2 | 19.6 | 59.6 |
| Polychaetes | 0.5 | 0.2 | 8.6 | 1.4 | 13.2 | 72.8 |
|  |  |  |  |  |  |  |
| *Control* | Group FER | Group IUI |  |  |  |  |
| Polychaetes | 0.6 | 0.2 | 24.5 | 1.3 | 33.0 | 33.0 |
| Sponges | 0.5 | 0.6 | 24.3 | 1.3 | 32.7 | 65.7 |
| Corals | 0.3 | 0.1 | 11.8 | 1.1 | 15.9 | 81.5 |
|  |  |  |  |  |  |  |
| *Undersides* |  |  |  |  |  |  |
| Group | Average Abundance | Average Abundance | Average Dissimilarity | Dissimilarity/SD | Contributing % | Cumulative % |
| *FER* | Group E | Group C |  |  |  |  |
| Sponges | 1.5 | 0.5 | 8.1 | 1.8 | 31.5 | 31.5 |
| Ascidians | 2.4 | 1.4 | 8.0 | 1.6 | 31.0 | 62.5 |
| Bivalves | 1.9 | 2.3 | 3.3 | 1.4 | 12.7 | 75.2 |
|  |  |  |  |  |  |  |
| *IUI* | Group E | Group C |  |  |  |  |
| Ascidians | 1.4 | 0.2 | 16.7 | 2.0 | 37.0 | 37.0 |
| Bivalves | 0.7 | 0.8 | 7.4 | 1.1 | 16.1 | 53.1 |
| Sponges | 0.4 | 0.1 | 5.9 | 1.4 | 12.8 | 65.9 |
| Polychaetes | 0.6 | 0.4 | 5.0 | 1.3 | 10.9 | 76.7 |
|  |  |  |  |  |  |  |
| *Exclusion* | Group FER | Group IUI |  |  |  |  |
| Bivalves | 1.9 | 0.7 | 10.6 | 2.1 | 24.9 | 24.9 |
| Sponges | 1.5 | 0.4 | 9.6 | 2.0 | 22.4 | 47.4 |
| Ascidians | 2.4 | 1.4 | 9.4 | 1.2 | 21.9 | 69.3 |
| Bryozoans | 1.4 | 0.7 | 5.6 | 2.3 | 13.2 | 82.5 |
|  |  |  |  |  |  |  |
| *Control* | Group FER | Group IUI |  |  |  |  |
| Bivalves | 2.3 | 0.8 | 18.4 | 2.5 | 35.3 | 35.3 |
| Ascidians | 1.4 | 0.2 | 12.7 | 2.0 | 24.4 | 59.7 |
| Polychaetes | 0.9 | 0.4 | 5.2 | 1.6 | 10.0 | 69.7 |
| Bryozoans | 1.0 | 0.7 | 5.2 | 1.3 | 10.0 | 79.6 |
|  |  |  |  |  |  |  |
