# Supplementary material for: Benthic community succession on artificial and natural coral reefs in the northern Gulf of Aqaba, Red Sea
Source: PLoS One. 2019 Feb 27;14(2):e0212842. doi: 10.1371/journal.pone.0212842 (PMC6392313; doi:10.1371/journal.pone.0212842)
Supplement: S11 Table — Data are feeding frequency (bites min-1) for all fish species on collector topsides and undersides at a suspended artificial reef (FER), a seafloor artificial reef (IGL), and at a natural reef (IUI) based on video records from 29 March and 19 April 2016 (S1 Table). Data are mean ± SE for video records (n) in which fish were observed biting the collectors. (DOCX) [file pone.0212842.s015.docx]

**S11 Table.**

| Site | n | Mean ± SE |
| --- | --- | --- |
| *Topsides* |  |  |
| FER | 15 | 0.8 ± 0.4 |
| IGL | 9 | 0.2 ± 0.2 |
| IUI | 17 | 0.3 ± 0.2 |
|  |  |  |
| *Undersides* |  |  |
| FER | 15 | 0 |
| IGL | 9 | 0 |
| IUI | 17 | 0.5 ± 0.3 |
