# Supplementary material for: Benthic community succession on artificial and natural coral reefs in the northern Gulf of Aqaba, Red Sea
Source: PLoS One. 2019 Feb 27;14(2):e0212842. doi: 10.1371/journal.pone.0212842 (PMC6392313; doi:10.1371/journal.pone.0212842)
Supplement: S12 Table — Species identification and observed behaviour on collector topsides and undersides at 3 sites in the mensurative experiment (artificial reefs at FER and IGL, natural reef at IUI) from all video records. Species: Scarus fuscopurpureus, Scarus ferrugineus, Ctenochaetus striatus, Acanthurus nigrofuscus, Zebrasoma xanthurum, Parupeneus forsskali, Heniochus diphreutes, Cyclichthys spilostylus, Ecsenius gravieri, Neopomacentrus miryae, Neopomacentrus xanthurus, Dascyllus trimaculatus, Pseudanthias squamipinnis. Behaviour: biting (B), foraging (F), egg aeration (A), defending territory (D), sheltering (S). (DOCX) [file pone.0212842.s016.docx]

**S12 Table**

|  | FER | IGL | IUI |
| --- | --- | --- | --- |
|  |  |  |  |
| *Topsides* | *D. trimaculatus*: B,A,D | *S. ferrugineus:* B | *S. fuscopurpureus*: B |
|  | *N. miryae*: B,A,D | *C. striatus*: B | *A. nigrofuscus*: B |
|  |  | *Z. xanthurum*: B | *P. forsskali*: F |
|  |  | *D. trimaculatus*: A,D |  |
|  |  |  |  |
| *Undersides* | *D. trimaculatus*: S | *N. xanthurus*: S | *S. fuscopurpureus*: B |
|  | *N. miryae*: S | *E. graviera*: S | *C. striatus*: B |
|  | *P. squamipinnis*: S |  | *P. forsskali*: F |
|  |  |  | *H. diphreutes*: S |
|  |  |  | *C spilostylus*: S |
